# A role for ErbB signaling in the induction of reactive astrogliosis

**DOI:** 10.1038/celldisc.2017.44

**Published:** 2017-12-05

**Authors:** Jing Chen, Wanwan He, Xu Hu, Yuwen Shen, Junyan Cao, Zhengdong Wei, Yifei Luan, Li He, Fangdun Jiang, Yanmei Tao

**Affiliations:** 1 Institute of Life Sciences, College of Life and Environmental Sciences, Hangzhou Normal University, Hangzhou, China; 2 Key Lab of Organ Development and Regeneration of Zhejiang Province, Hangzhou, China; 3 Key Lab of GEM Resource and Model Research of Hangzhou, Hangzhou, Zhejiang, China

**Keywords:** receptor tyrosine kinase, glia, gliosis, brain injury, anorexia

## Abstract

Reactive astrogliosis is a hallmark of many neurological disorders, yet its functions and molecular mechanisms remain elusive. Particularly, the upstream signaling that regulates pathological responses of astrocytes is largely undetermined. We used a mouse traumatic brain injury model to induce astrogliosis and revealed activation of ErbB receptors in reactive astrocytes. Moreover, cell-autonomous inhibition of ErbB receptor activity in reactive astrocytes by a genetic approach suppressed hypertrophic remodeling possibly through the regulation of actin dynamics. However, inhibiting ErbB signaling in reactive astrocytes did not affect astrocyte proliferation after brain injury, although it aggravated local inflammation. In contrast, active ErbB signaling in mature astrocytes of various brain regions in mice was sufficient to initiate reactive responses, reproducing characterized molecular and cellular features of astrogliosis observed in injured or diseased brains. Further, prevalent astrogliosis in the brain induced by astrocytic ErbB activation caused anorexia in animals. Therefore, our findings defined an unrecognized role of ErbB signaling in inducing reactive astrogliosis. Mechanistically, inhibiting ErbB signaling in reactive astrocytes prominently reduced Src and focal adhesion kinase (FAK) activity that is important for actin remodeling, although ErbB signaling activated multiple downstream signaling proteins. The discrepancies between the results from loss- and gain-of-function studies indicated that ErbB signaling regulated hypertrophy and proliferation of reactive astrocytes by different downstream signaling pathways. Our work demonstrated an essential mechanism in the pathological regulation of astrocytes and provided novel insights into potential therapeutic targets for astrogliosis-implicated diseases.

## Introduction

Astrocytes, the principal macroglial cells in the central nervous system (CNS), are fundamental to brain function and health [1, 2]. In pathological conditions, astrocytes react to almost all forms of CNS insults. Astrocytes’ pathological responses typically involve increased expression of glial fibrillary acidic protein (GFAP), cellular hypertrophy and enhanced proliferation. This pattern is indicative of reactive astrogliosis, a hallmark of diverse neurological disorders including trauma, ischemia, neurodegeneration, multiple sclerosis, epilepsy and autism [3–5]. A growing body of evidence indicates that reactive astrocytes are not bystanders during disease development [5]. However, their functions and regulatory mechanisms remain elusive.

Reactive astrocytes responding to pathological insults are distinct from normal astrocytes in morphology, functions and molecular profiles [1, 5–7]. To explore these astrocytes’ regulatory mechanisms, many molecules have been investigated over the past few decades. However, many of them exhibit paradoxical implications [5–7]. Further complicating the picture is the fact that the regulatory mechanisms of reactive gliosis have been most frequently studied in cell culture models. Findings from such models are increasingly being tested in genetically engineered mice, with some studies failing to confirm *in vitro* findings *in vivo*. For example, cytoplasmic or nuclear signaling proteins, such as Akt, mechanistic target of rapamycin, extracellular signal-regulated kinase (Erk) and signal transducer and activator of transcription 3 (STAT3), are upregulated in reactive astrocytes, and their positive involvement in regulating astrogliosis has been confirmed by genetic approaches *in vivo* [8–14]. Receptor tyrosine kinases are upstream activators of these signaling pathways [15–17]. Among these, fibroblast growth factor 2 (FGF2) and its receptor (FGFR) increase in reactive astrocytes, promote GFAP expression in cultured astrocytes, and are thus thought to mediate astrocytes’ reactive responses. Surprisingly, recent loss- and gain-of-function studies on genetically engineered mice show that FGF signaling inhibits astrocyte reactivity under both uninjured and injured conditions [18]. These findings emphasize that the upstream signaling that regulates astrogliosis remains largely undetermined.

ErbB receptors (ErbB1–4), another family of receptor tyrosine kinases, and their ligands, including epidermal growth factor (EGF), neuregulin (NRG) and transforming growth factor α (TGFα), have been reported to increase in tissues with reactive astrogliosis [19–21]. In the nervous system, ErbB receptors are differentially expressed across various neural cell types and regulate many developmental and pathological events [16, 17, 22]. Once ligand bound, ErbB receptors dimerize and activate multiple intracellular signaling pathways, including Akt/mechanistic target of rapamycin, Erk and STAT3, to potently regulate cell proliferation, survival, differentiation, migration and inflammatory responses [16, 17, 22]. Both mutations and post-transcriptional alteration of ErbB receptors have been implicated in neurological disorders, including demyelination, stroke, epilepsy and psychiatric disorders [16, 22]. However, it remains unknown whether aberrant ErbB signaling in astrocytes participates in disease progression.

To examine whether ErbB signaling has a role in astrocytes of injured or diseased brains, we manipulated ErbB receptor activity in mature astrocytes by adopting a pan-ErbB strategy in mice. By conditionally expressing either a dominant-negative ErbB mutant that inhibited any ErbB receptor when coexpressed in the same cell or a constitutively active ErbB mutant that promoted ErbB receptor activation, we circumvented the limited information on ErbB receptor composition in astrocytes and focused on the function of ErbB signaling. Through *in vivo* studies combining loss- and gain-of-function approaches, we found that ErbB signaling positively regulated astrocyte reactivity, exerting a direct effect on hypertrophic remodeling and a cooperative effect on other features of reactive astrocytic responses.

## Results

### Specific inhibition of ErbB receptor activity in reactive astrocytes *in vivo*

*In situ* hybridization studies have revealed that the epidermal growth factor receptor (EGFR/ErbB1) and ErbB2 are expressed in astrocytes [23]. After non-detection of ErbB receptors in astrocytes of the adult brain by immunostaining, we generated *Mlc1*-tTA;*TRE*-dnEGFR (*Mlc1*-dnEGFR) bi-transgenic mice by crossing *Mlc1*-tTA mice with *TRE*-dnEGFR mice to investigate the existence of functional ErbB signaling in mature astrocytes. The *Mlc1* gene encodes megalencephalic leukoencephalopathy with subcortical cysts-1 (Mlc1), a membrane protein expressed specifically in GFAP-positive cells in the adult brain [24]. Thus, in our transgenic mice, the *Mlc1* promoter drove the expression of tetracycline-controlled transactivator (tTA) in astrocytes to activate *tetracycline-responsive element* (*TRE*)*-*controlled transcription through a ‘Tet-off’ system [24].

**Figure 1:**
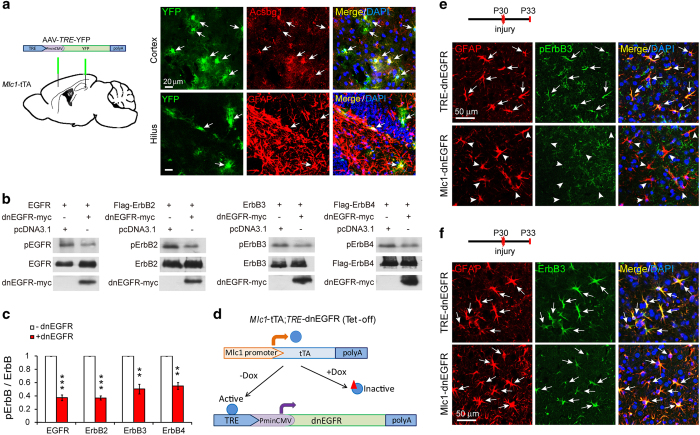
Astrocyte-specific expression of dnEGFR blocked injury-induced ErbB activation in reactive astrocytes. (**a**) Representative images of *TRE*-YFP expression in astrocytes of *Mlc1*-tTA mice at 1-month old. AAV-*TRE*-YFP was stereotaxically injected into indicated brain regions. Fixed brains collected 1 day later were sectioned and immunostained for Acsbg1 or GFAP to label astrocytes in the cerebral cortex or hippocampal hilus, respectively. White arrows, double-positive cells. (**b**) Efficient inhibition on activity of each ErbB receptor by dominant-negative mutant dnEGFR. Complementary DNA sequence of dnEGFR was amplified from genomic DNA of *TRE*-dnEGFR mice by PCR and cloned into pcDNA3.1-His/myc vector. pcDNA3.1-dnEGFR-myc was transfected into HEK293 cells by polyethylenimine (PEI) together with one of ErbB1–4 plasmids or an empty vector. Cells were lysed 24 h later and processed into WB with indicated antibodies. Any ErbB receptor when overexpressed in HEK293 cells would autophosphorylate itself independent of ligand stimulation. Shown are representative WB results, demonstrating the inhibition of dnEGFR on phosphorylation of each ErbB receptor. (**c**) Quantitative analyses of experiments in (**b**). ***P<0.001; **P<0.01; *n*=3 for each ErbB receptor, paired *t*-test. (**d**) Schematic illustration of the Tet-off system in *Mlc1*-tTA;*TRE*-dnEGFR (*Mlc1*-dnEGFR) mice. (**e**, **f**) Active ErbB3 (pErbB3), but not total ErbB3 levels, was suppressed in the reactive astrocytes of *Mlc1*-dnEGFR cortex. Cortical slices from indicated mice 3 days post injury were immunostained with antibodies against pErbB3 (**e**) or total ErbB3 (**f**) together with GFAP. White arrows, double positive cells. Arrowheads, cells positive for GFAP alone.

To confirm the specificity of *Mlc1*-tTA, an adeno-associated virus (AAV) harboring a *TRE*-yellow fluorescent protein (YFP) reporter was stereotaxically injected into the brain of 1-month-old *Mlc1*-tTA mice (Figure 1a). To avoid confusion with a reactive response, *Mlc1*-tTA targeting cells in the normal brain were identified by YFP fluorescence 1 day after virus injection. YFP-positive (YFP^+^) cells in the hippocampal hilus were immunopositive for GFAP (Figure 1a). It is now well known that astrocytes in the brain are heterogeneous. Although GFAP, an intermediate filament protein, is a reliable marker for astrocytes in the white matter, cerebellum and hippocampus of the adult brain, it is normally undetectable in the cerebral cortex [7, 11, 25]. Therefore, to confirm the specificity of YFP^+^ cells in the cerebral cortex, we immunostained AAV-injected cortical slices with an antibody to Acyl-CoA synthetase bubblegum family member 1 (Acsbg1), a protein marker for a wider spectrum of astrocytes [11, 26]. YFP^+^ cells in the cortex were positive for Acsbg1 (Figure 1a). In addition to adult astrocytes, *Mlc1*-tTA also targeted astrocyte-like adult neural stem cells (NSCs), as indicated by the presence of YFP-labeled cells coexpressing the NSC marker, nestin (Supplementary Figure 1a and b). Nevertheless, nestin^+^ cells in the normal adult brain were strictly localized in NSC niches, such as the subgranular zone, subcorpus callosum zone and subventricular zone.

After confirming the specific targeting of *Mlc1*-tTA to astrocytes in the cortex, we employed *Mlc1*-dnEGFR double transgenic mice to study ErbB receptor function in astrocytes. *TRE*-dnEGFR transgenic mice encode a dominant-negative mutant of EGFR (dnEGFR) that is ectopically expressed under the control of a *TRE* with a cytomegalovirus minimal promoter (*PminCMV*) [27]. DnEGFR is a truncated form of EGFR that lacks the intracellular kinase domain but retains the ability to bind to other ErbB family members [27]. In this study, efficient suppression of ErbB receptor activity by dnEGFR was first verified by coexpressing dnEGFR with a comprehensive panel of ErbB receptors in HEK293 cells. When coexpressed with dnEGFR, the activity of each ErbB receptor was reduced 50–60%, indicating haploinsufficient function (Figure 1b and c). When not fed the tetracycline analog doxycycline (Dox), bi-transgenic *Mlc1*-dnEGFR mice expressed dnEGFR in *Mlc1*-controlled cells (Figure 1d). After failing to detect dnEGFR with available antibodies, we examined dnEGFR transcripts through real-time reverse transcription polymerase chain reaction (RT-PCR) and observed a 20- to 60-fold increase in the cerebral cortices of *Mlc1*-dnEGFR mice compared with those of littermate controls (Supplementary Figure S2a and b). Variations in the increase of dnEGFR transcripts in different *Mlc1*-dnEGFR mice may have been caused by varied insertion copies of the ectopic gene. Notably, dnEGFR expression in astrocytes during development did not change animal weight, body size or gross brain structures. In addition, immunostaining of GFAP or Acsbg1 in brain slices did not reveal significant differences in astrocyte number, distribution or size (Supplementary Figure S2c–g), suggesting that reduced ErbB receptor activity did not alter astrocyte development in *Mlc1*-dnEGFR mice.

To investigate whether ErbB signaling is required for reactive astrogliosis, we used an acute brain injury model in which the parietal cortices of mice, aged postnatal day 30 (P30), were exposed and injured via a needle puncture. Three to 21 days later, brains were isolated, sectioned and immunostained for GFAP. Increased GFAP expression is a clear hallmark indicating the transition of normal astrocytes to reactive astrocytes [5–7]. Both *Mlc1*-dnEGFR and littermate control mice exhibited increased GFAP^+^ cells adjacent to the injury sites, with comparable distributions (Supplementary Figure S3a). Reactive astrocytes possess a molecular profile distinct from that of normal astrocytes [5, 7, 28]. For example, nestin, the protein marker for NSCs, appears to be a molecular hallmark of reactive astrocytes induced by various insults [29, 30], and it appeared in reactive astrocytes in either *Mlc1*-dnEGFR or control cortices 3 days after injury (Supplementary Figure S3b).

Using this model, we observed similar increases in dnEGFR transcripts in injured and intact *Mlc1*-dnEGFR cortices (Supplementary Figure S3c and d). To investigate whether ErbB receptor activity was altered in the reactive astrocytes of *Mlc1*-dnEGFR mice, injured cortical slices were costained with antibodies to GFAP and the active forms of ErbB1–4. Although other antibodies did not detect signals, immunoreactivity of phosphorylated ErbB3 (pErbB3) was clearly observed in the reactive astrocytes of control mice (Figure 1e). Notably, pErbB3 was hardly detected in the reactive astrocytes of *Mlc1*-dnEGFR mice, indicating inhibition of endogenous ErbB signaling by dnEGFR (Figure 1e). Note non-detection of pErbB3 was not due to loss of the ErbB3 protein because ErbB3 immunoreactivity was present in the reactive astrocytes of *Mlc1*-dnEGFR mice in a manner similar to that observed in controls (Figure 1f). The involvement of other ErbB receptors in reactive astrocytes could not be excluded, as their absence could have been due to the used assays being insensitive to small amounts of protein. In order to test this idea, we cultured primary astrocytes isolated from *TRE*-dnEGFR or *Mlc1*-dnEGFR mouse brains. All four ErbB receptors were detected in the primarily cultured astrocytes by Western blotting (WB) (Supplementary Figure S3e). We treated the primary astrocytes with recombinant human EGF (rhEGF), a ligand specifically binding to EGFR, and revealed activation of all four ErbB receptors in control astrocytes (Supplementary Figure S3e and f). Unsurprisingly, ErbB receptor activities induced by rhEGF were significantly reduced in primary *Mlc1*-dnEGFR astrocytes (Supplementary Figure S3f). Treatment with recombinant human NRG1, another ligand that specifically binds to ErbB3 or ErbB4, induced ErbB4 activation in primary control astrocytes. Remarkably, it was significantly reduced in primary *Mlc1*-dnEGFR astrocytes (Supplementary Figure S3e and f). These results consolidated the inhibitory effects of dnEGFR on ligand-induced ErbB receptor activation. It was noticeable that basic activities of EGFR, ErbB2 and ErbB3 were suppressed in primary *Mlc1*-dnEGFR astrocytes (Supplementary Figure S3f), suggesting an inhibition on efficiency of endogenous ErbB ligands. Therefore, dnEGFR targeting of ErbB receptor kinase activity helped us circumvent the limited knowledge of ErbB receptor composition *in vivo* and focus on characterizing their function in reactive astrocytes.

### Inhibition of ErbB signaling in reactive astrocytes suppressed their morphological expansion

**Figure 2:**
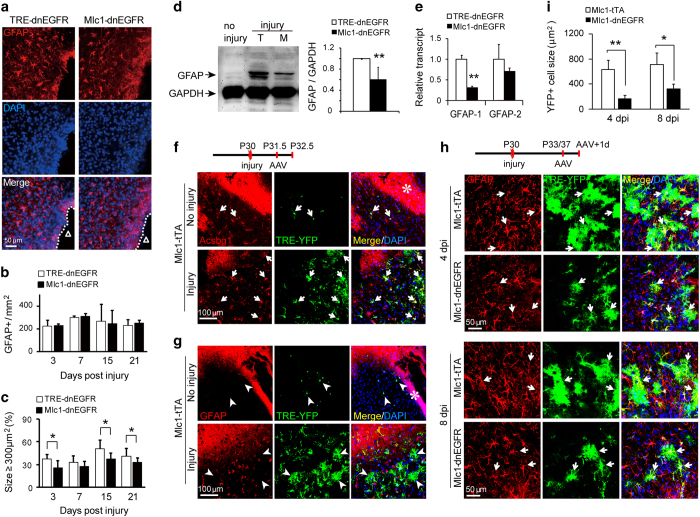
Inhibition of endogenous ErbB signaling suppressed hypertrophic expansion of reactive astrocytes. (**a**) GFAP immunostaining of cortices 3 days post injury showed there were many GFAP^+^ cells in *Mlc1*-dnEGFR cortices smaller than those in littermate controls. ‘Δ’ represents the injury sites. (**b**) Quantitative analyses of GFAP^+^ cell densities near the injury sites in the cortices of *Mlc1*-dnEGFR and littermate control mice on different days post injury. *n*=3 for each time point, paired *t*-test. (**c**) Quantitative analyses of the percentage of GFAP^+^ cells induced by injury with GFAP-immunoreactive area bigger than 300 μm^2^. *n*=3 for each time point, and over 600 cells were analyzed for each mouse. *P<0.05, paired* t-*test. (**d**) Less increase of GFAP protein in injured cortical tissues from *Mlc1*-dnEGFR mice (M) in comparison with that from littermate *TRE*-dnEGFR controls (T) 3 days post injury. Shown are representative WB result (left) and quantitative analysis result (right, **P=0.002, *n*=4 for each group, paired* t-*test). (**e**) Transcription of GFAP isoform 1 was specifically suppressed in injured *Mlc1*-dnEGFR cortices. Total RNA was extracted from injured cortical tissues of *Mlc1*-dnEGFR and littermate mice. mRNA levels of GFAP isoform 1 and isoform 2 were evaluated by real-time RT-PCR with specific primers. **P=0.001, *n*=3, paired* t-*test. (**f**) Hypertrophy labeled by diffused YFP in reactive astrocytes induced by stab injury. Injured or uninjured cortices of *Mlc1*-tTA mice were stereotaxically injected with AAV-*TRE*-YFP on 1.5 day post injury, and collected 1 day later for immunostaining of non-selective astrocyte marker Acsbg1. All YFP-labeled (YFP^+^) cells were positive for Acsbg1 (white arrows). Note YFP^+^ cells in the injured cortex were hypertrophic and easy to be observed, whereas that in the uninjured cortex were small and hardly observed in the area farther from the injection sites. The asterisk indicates the needle track for viral injection. (**g**) Hypertrophic remodeling was independent of GFAP expression in cortical astrocytes in response to injury. Similar samples from experiments in (**f**) were immunostained for GFAP. Note many reactive astrocytes with hypertrophy exhibited no GFAP signal (white arrowheads). (**h**) YFP-labeled sizes of reactive astrocytes were strikingly reduced in the injured cortices of *Mlc1*-dnEGFR mice. Brain-injured *Mlc1*-dnEGFR and *Mlc1*-tTA mice were stereotaxically injected with AAV-*TRE*-YFP near the injury sites on 3 or 7 days post injury (dpi) and brains were collected 1 day later for immunostaining of GFAP. Note all YFP^+^ cells were positive for GFAP at these time points (white arrows). Images were taken within 300-μm areas near the injury sites. (**i**) Quantitative analyses of YFP-labeled cell sizes induced by stab injury in *Mlc1*-dnEGFR mice and littermate *Mlc1*-tTA controls. *n*=5 for each time point. **P=0.008 for 4, and *P=0.028 for 8, days post injury (dpi), paired* t-*test.

In addition to increased expression of GFAP, reactive astrocytes exhibit cellular hypertrophy. We found that most GFAP^+^ cells in the injured cortices of *Mlc1*-dnEGFR mice were smaller than those in control mice (Figure 2a). Quantitative analysis showed that despite a similar distribution of GFAP^+^ cells (Figure 2b), reactive astrocytes with a GFAP-immunoreactive area larger than 300 μm^2^ were significantly reduced in *Mlc1*-dnEGFR cortices at several time points after injury (Figure 2c). Consistently, WB showed that the increase in GFAP protein levels in injured cortical tissues from *Mlc1*-dnEGFR mice was 60.74±23.5% of that from littermate controls (Figure 2d). Reduction of GFAP was caused by an inhibition on GFAP gene transcription, and mRNA level of *gfap* splicing isoform 1 was significantly reduced (Figure 2e).

Dual mutation of GFAP and vimentin, a fellow intermediate filament protein, causes a small reduction in the extension of cellular processes of reactive astrocytes, whereas normal astrocytes are not affected [31]. These proteins are thought to be effectors mediating hypertrophic remodeling. However, we found that the morphological expansion of astrocytes was independent of GFAP expression during injury-induced reactive responses. Injection of AAV-*TRE*-YFP into injured *Mlc1*-tTA cortices caused reactive astrocytes to be labeled with YFP fluorescence (Figure 2f). Specificity of YFP-labeled astrocytes and reactive astrocytes was further verified by co-labeling with antibodies against glutamine synthetase (GS) and nestin, respectively (Supplementary Figure S4a and b). Two and a half days after injury, YFP^+^ cells were hypertrophic and positive for Acsbg1 (Figure 2f). GFAP did not appear at this stage in every reactive astrocyte, whereas all YFP-labeled astrocytes in the injured cortices exhibited expanded sizes (Figure 2g). Especially, many hypertrophic astrocytes expressed little GFAP (Figure 2g, white arrowheads). Thus, morphological remodeling was likely a reactive response that preceded the onset of GFAP protein expression in astrocytes.

To confirm that YFP distribution represented completely the morphology of astrocytes, we used the method that was described previously [31, 32], and injected Alexa Fluor 568, which has smaller molecular weight into YFP-labeled cells in fixed brain slices. As shown in Supplementary Figure S5a and b, both Alexa Fluor 568 and YFP labeled astrocytic cell bodies and fine processes. However, because of labeling the cell at living status, YFP distribution exhibited more complete morphology than the injected fluorescent dye did (Supplementary Figure S5a and b). Therefore, YFP labeling would reliably help us to evaluate the astrocytic sizes in mouse cortices. Four days after injury, more reactive astrocytes in the injured cortices of both *Mlc1*-dnEGFR and littermate mice expressed detectable GFAP, as all cells targeted by AAV-*TRE*-YFP were immunopositive for GFAP (Figure 2h). Suppression of hypertrophy in reactive astrocytes in *Mlc1*-dnEGFR cortices was more striking when cell sizes were measured according to the distribution of YFP fluorescence, in comparison with that based on GFAP immunoreactivity (Figure 2h). Four and 8 days after injury, the YFP-labeled reactive astrocytes in *Mlc1*-dnEGFR cortices exhibited sizes 28.06±10.3% and 47.30±14.6%, respectively, of those in littermate controls (Figure 2i). Taken together, these results suggested that the morphological reaction of astrocytes was more likely to depend on actin remodeling than intermediate filament extension. Further, these findings revealed that the regulation of this hypertrophic remodeling was ErbB dependent.

### Inhibition of ErbB signaling in reactive astrocytes reduced scar thickness but did not affect cell proliferation

**Figure 3:**
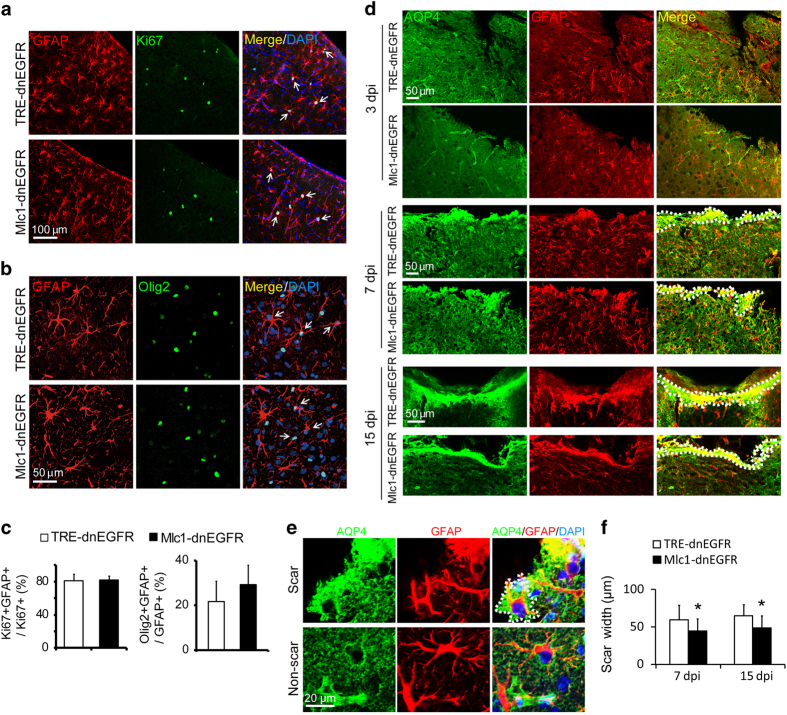
Inhibition of endogenous ErbB signaling in reactive astrocytes did not affect their proliferation but reduced scar thickness. (**a,** **b**) Comparable proliferation of reactive astrocytes in *Mlc1*-dnEGFR mice and littermate controls. Cortical slices from indicated mice 3 days post injury were immunostained for GFAP with either Ki67 (**a**) or Olig2 (**b**), respectively. White arrows, representative double positive cells. (**c**) Quantitative analyses of experiments in (**a**, **b**). *n*=3 for each group, paired* t-*test. (**d**) Representative images of scars formed by reactive astrocytes at the injury sites as shown by increased immunoreactivities of AQP4 and GFAP. Mouse cortices were stabbed by a blade parallel to the longitudinal fissure. Brains on 3, 7 or 15 days post injury (dpi) were isolated for sections crossing the injured grooves. Note there was no glial scar formed on 3 dpi. Dotted lines outlined the scar surface and the scar boundary. (**e**) AQP4 immunoreactivity shaped scar-forming astrocytes well. Injured cortices on 7 dpi were immunostained for AQP4 and GFAP. Note scar-forming astrocytes had abundant AQP4 immunoreactivity that exhibited more cell bodies and processes than GFAP immunoreactivity did, whereas AQP4 immunostaining in non-scar area failed to shape astrocytes. Dotted lines outlined a scar-forming astrocyte in wound surface. (**f**) The thickness of glial scars formed in the cortices of *Mlc1*-dnEGFR mice was reduced as compared with that formed in littermate controls 7 or 15 days post injury (dpi). Scar width was measured from the wound surface to the scar boundary according to the immunoreactivities of AQP4 plus GFAP. *P<0.05, paired* t-*test, *n*=3 for each time point.

We also characterized the increased proliferation of reactive astrocytes. Some of the reactive astrocytes induced by stab injury in both *Mlc1*-dnEGFR and control mice were immunopositive for the proliferation marker Ki67 (Figure 3a). In addition, reactive astrocytes were frequently labeled with Olig2 immunostaining (Figure 3b). The basic helix-loop-helix transcription factor Olig2 has recently been identified as a protein hallmark of astrogliosis [33], despite being a key factor in determining motor neuron and, subsequently, oligodendroglial fate in NSCs during embryonic development [34]. In the normal brain, the majority of Olig2^+^ nuclei, which indicate oligodendroglial lineage, are distributed in the white matter, with only sparse localization of Olig2^+^ cells in the cerebral cortex. Notably, increased Olig2^+^ nuclei in the parenchyma have been discovered in stab-, freeze-, ischemia- or Aβ deposit-injured cerebral cortical tissues [33, 35, 36], with many localized in reactive astrocytes [33, 36]. Lineage tracing studies have demonstrated that Olig2^+^ astroglial cells are an essential source of increased reactive astrocytes in response to injury [33]. Indeed, some Olig2^+^ nuclei were also positive for Ki67 in the stab-injured cortex (Supplementary Figure S6). Surprisingly, neither the proportion of Ki67^+^ nor that of Olig2^+^ reactive astrocytes were changed in *Mlc1*-dnEGFR mice (Figure 3c), suggesting that dnEGFR did not affect their proliferation.

Acute injury induces proliferation of reactive astrocytes and extensive intermingling of their elongated processes, forming glial scars surrounding damaged CNS tissues [37]. Although glial scars limit damage in tissues, they may elicit seizures in patients after brain injury [38]. Many factors generated by different cell sources influence scar formation [39]. We employed a more severe injury model by stabbing mouse cortices with a sharp blade, and examined scars formed 7 and 15 days after injury (Figure 3d). Scar thickness was evaluated according to the immunoreactivity of GFAP plus that of AQP4, a membrane protein whose immunoreactivity labeled the full mormorphology of scar-forming astrocytes (Figure 3d and e). We found that astrocytic scars in the injured cortices of *Mlc1*-dnEGFR mice were formed thinner than those in littermate controls (Figure 3d and f). Thus, with the suppression on morphological expansion, scar formation ability of reactive astrocytes was impaired in *Mlc1*-dnEGFR mice.

### Ectopically activated ErbB signaling in astrocytes induced comprehensive features of reactive astrogliosis

The loss-of-function studies revealed that ErbB signaling was specifically required for hypertrophic remodeling of reactive astrocytes. To examine whether ErbB activation in astrocytes was solely responsible for the hypertrophic response, we generated another mouse line with inducible gene expression in astrocytes by crossing *Mlc1*-tTA with *TRE*-ErbB2^V664E^ mice. Among ErbB1–4 receptors, ErbB2 does not bind any known ligand but is the preferred partner of other ErbB members [40]. Moreover, dimerization with ErbB2 potentiates the downstream signaling of ErbB receptors [16]. The ectopically expressed gene in *TRE*-ErbB2^V664E^ transgenic mice encodes an active form of rat ErbB2 (ErbB2^V664E^) that contains an amino-acid mutation (Vla_664_/Glu_664_) within the transmembrane domain that facilitates its dimerization and activation [41].

**Figure 4:**
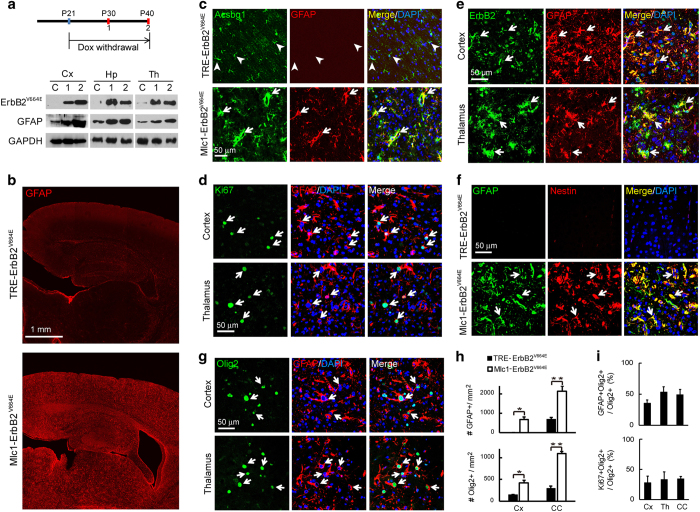
Ectopically activated ErbB signaling in astrocytes throughout the brain induced reactive responses comprehensively. (**a**) ErbB2^V664E^ induction in *Mlc1*-ErbB2^V664E^ mice by Dox withdrawal according to the illustrated timetable. Mice were fed with Dox from embryonic stages, and killed to isolate different brain regions on P40 (C, no Dox withdrawal; 2, Dox withdrawal from P21 to P40), or P30 (1, Dox withdrawal from P21 to P30), respectively, for WB. Note GFAP increased concurrently with ErbB2^V664E^. (**b**) GFAP immunoreactivity increased prevalently in the brain of *Mlc1*-ErbB2^V664E^ mice 20 days after Dox withdrawal. (**c**) GFAP and Acsbg1 colocalized in cortical astrocytes of *Mlc1*-ErbB2^V664E^ mice. Cortical slices from *Mlc1*-ErbB2^V664E^ and littermate mice after Dox withdrawal were immunostained for GFAP and Acsbg1. Arrowheads, cells positive for Acsbg1 alone. White arrows, double positive and hypertrophic cells. (**d**) Many GFAP^+^ cells were positive for Ki67 in the brain of *Mlc1*-ErbB2^V664E^ mice. White arrows, GFAP^+^Ki67^+^ cells. A nucleus (DAPI^+^) for a GFAP^+^ cell was identified by the association with its main cell body. (**e**) ErbB2^V664E^ was well localized in GFAP^+^ cells in the brain of *Mlc1*-ErbB2^V664E^ mice. White arrows, GFAP^+^ErbB2^+^ cells. (**f**) Expression of nestin in GFAP^+^ cells in the cortices of *Mlc1*-ErbB2^V664E^ mice. White arrows, GFAP^+^nestin^+^ cells. (**g**) Many GFAP^+^ cells had Olig2^+^ nuclei in the brain of *Mlc1*-ErbB2^V664E^ mice. White arrows, GFAP^+^Olig2^+^ cells. A nucleus (DAPI^+^) for a GFAP^+^ cell was identified by the association with its main cell body. (**h**) Quantitative analyses of GFAP^+^ or Olig2^+^ cell densities in the Cx or CC in *Mlc1*-ErbB2^V664E^ and littermate control mice. *P<0.05; **P<0.01; *n*=3 for each brain region, paired* t-*test. (**i**) Quantitative analyses of the percentage of GFAP^+^Olig2^+^ or Ki67^+^Olig2^+^ cells in Olig2^+^ cells in different brain regions of *Mlc1*-ErbB2^V664E^ mice. *n*=3 for each brain region. Cx, cortex; Th, thalamus; CC, corpus callosum; Hp, hippocampus.

To generate this line, *TRE*-ErbB2^V664E^ mice were mated with *Mlc1*-tTA mice. Pregnant mice and their offspring were fed with Dox until weaning occurred at P21, whereas bi-transgenic *Mlc1*-tTA;*TRE*-ErbB2^V664E^ (*Mlc1*-ErbB2^V664E^) mice and littermate controls were allowed to grow to adulthood. Feeding the bi-transgenic *Mlc1*-ErbB2^V664E^ mice with Dox blocked ErbB2^V664E^ expression, while withdrawal of Dox initiated it (Figure 4a). It is notable that ErbB2^V664E^ expression induced by Dox withdrawal was accompanied by an increase in GFAP in a time-dependent manner (Figure 4a). Immunostaining showed that GFAP^+^ cells increased dramatically throughout the brain 20 days after Dox withdrawal (Figure 4b).

To confirm that the detected GFAP^+^ cells were indeed astrocytes in the cerebral cortex, we costained the cortical slices with antibodies to GFAP and Acsbg1. Acsbg1 immunoreactivity was morphologically reminiscent of the staining observed in the cortical astrocytes of control mice and colocalized well with increased GFAP in *Mlc1*-ErbB2^V664E^ cortices (Figure 4c). Moreover, Acsbg1^+^GFAP^+^ astrocytes possessed enlarged cell bodies and broadened cellular processes, indicating a hypertrophy (Figure 4c). Remarkably, the increased number of GFAP^+^ astrocytes was due to both GFAP expression in normally GFAP-negative astrocytes and active astrocyte proliferation, as indicated by about half (60.95±4.00% in the cortex, 53.16±4.96% in the corpus callosum and 59.38±12.7% in the midbrain) of GFAP^+^ cells being immunopositive for Ki67 (Figure 4d).

The increased GFAP expression, hypertrophy and elevated proliferation of mature astrocytes implied the induction of spontaneous astrogliosis in *Mlc1*-ErbB2^V664E^ mice after Dox withdrawal. Consistent with the ectopic expression of ErbB2^V664E^, various brain regions in *Mlc1*-ErbB2^V664E^ mice exhibited immunoreactivity with the ErbB2 antibody (Supplementary Figure S7). This immunoreactivity localized well in GFAP^+^ cells, confirming cell-targeting specificity in *Mlc1*-ErbB2^V664E^ mice (Figure 4e). There was neither scarring nor aggregation of reactive astrocytes in the cerebral cortices, consistent with the idea that astrocytes in *Mlc1*-ErbB2^V664E^ mice reacted to intrinsic factors instead of extrinsic ones released from injury sites or degenerative plaques. To further test this idea, primary astrocytes purified from *Mlc1*-ErbB2^V664E^ and control mice were examined *in vitro*. Cultured cortical astrocytes from either control or *Mlc1*-ErbB2^V664E^ mice exhibited reactive features such as the GFAP expression (Supplementary Figure S8a). However, normal cultured astrocytes appeared to be flat and spreading, adhering well to the bottom of the cell culture dish, with only a few of them expressing the radial glial cell marker RC2, whereas astrocytes from *Mlc1*-ErbB2^V664E^ mice exhibited small but plump shapes and high levels of RC2, as well as accelerated proliferation rates (Supplementary Figure S8a and b). These results suggest that cell-autonomous ErbB activation promoted astrocyte proliferation. The observation of high RC2 expression in *Mlc1*-ErbB2^V664E^ astrocytes was in line with the previous report that TGFα stimulates cultured astrocytes to dedifferentiate into NSCs [42].

As another molecular hallmark for reactive astrocytes, nestin did not appear in the cerebral cortex in control mice, but was observed throughout the brain of *Mlc1*-ErbB2^V664E^ mice and colocalized well with GFAP^+^ cells (Figure 4f). Similarly, Olig2^+^ nuclei increased in various brain regions of *Mlc1*-ErbB2^V664E^ mice (Supplementary Figure S8c), with many localized in GFAP^+^ cells (Figure 4g). An increased proportion of Olig2^+^ cells was correlated with an increase in GFAP^+^ cells (Figure 4h). As in our experiments using the injury model, we observed that many Olig2^+^ cells were positive for Ki67 in the brains of *Mlc1*-ErbB2^V664E^ mice (Supplementary Figure S8d), indicating an actively proliferating status. Further, the percentage of Olig2^+^ cells positive for Ki67 was close to the percentage positive for GFAP (Figure 4i), suggesting that proliferative Olig2^+^ cells were mainly astrocytes in *Mlc1*-ErbB2^V664E^ mice. A similar colocalization pattern was observed for Olig2 and nestin in *Mlc1*-ErbB2^V664E^ mice (Supplementary Figure S8e). We examined the mRNA levels of a series of genes [43], and did not reveal a typical transcriptional pattern of subtype A1 or A2 for the reactive astrocytes in *Mlc1*-ErbB2^V664E^ mice (Supplementary Figure S8f). Both A1 and A2 subtype-specific genes were actively transcribed, suggesting reactive astrocytes induced in the brain of *Mlc1*-ErbB2^V664E^ mice were phenotypically heterogeneous.

Heterogeneity of reactive astrocytes might be caused by a complication of reactive microgliosis in *Mlc1*-ErbB2^V664E^ brain. In addition to effects on astrocytes, reactive astrogliosis in diseased or injured brains is always accompanied by recruitment of reactive microglia [39]. Indeed, we observed an increased prevalence of reactive microglia (Iba1^+^) in the brains of *Mlc1*-ErbB2^V664E^ mice comparable to that of GFAP^+^ cells (Supplementary Figure S9a and b). These results showed that active ErbB signaling in mature astrocytes induced reactive responses with a molecular and cellular profile similar to that observed in reactive astrogliosis in injured or diseased brains. Considering the lack of ectopic ErbB activation in microglia or leukocytes that were infiltrated through the blood–brain barrier (Supplementary Figure S9c), our findings suggest that reactive responses of microglia in *Mlc1*-ErbB2^V664E^ mice were induced by immunogenic factors released from reactive astrocytes. To confirm this idea, we examined *Mlc1*-ErbB2^V664E^ mouse brains 3 days after Dox withdrawal. At this early stage, GFAP-expressing astrocytes started to appear in the cortices of *Mlc1*-ErbB2^V664E^ mice, with some of them possessing Ki67^+^ nuclei. In contrast, Iba1^+^ cells in the same cortices did not increase, and did not have Ki67^+^ nuclei (Supplementary Figure S10a–d). These results were consistent with previous reports that reactive astrocytes release many factors including cytokines [28], and astrocytic gene targeting-induced astrogliosis results in reactive microglia responses [18]. Indeed, we detected increase of cytokines including TGF-β2, interleukin-6 (IL-6), IL-1β, C-C motif chemokine ligand 2 (CCL2), and Ciliary neurotrophic factor (CNTF) in primary *Mlc1*-ErbB2^V664E^ astrocytes (Supplementary Figure S10e). Noticeably, astrogliosis and microgliosis did not result in cell apoptosis. There were no terminal deoxynucleotidyl transferase-mediated deoxyuridine triphosphate nick-end labeling (TUNEL)^+^ cells revealed in *Mlc1*-ErbB2^V664E^ brain or littermate controls on either 3 or 20 days after Dox withdrawal (Supplementary Figure S10h).

### Prevalent astrogliosis in the brain induced by astrocytic ErbB activation resulted in anorexia in mice

**Figure 5:**
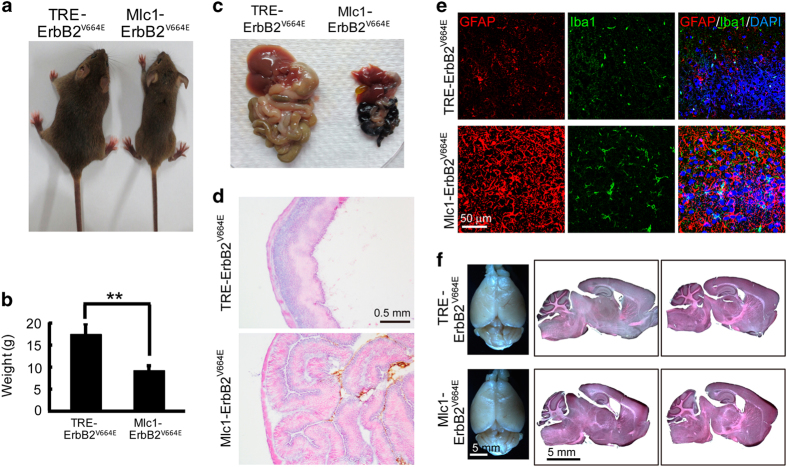
Prevalent astrogliosis and associated inflammation in the brain induced anorexia in *Mlc1*-ErbB2^V664E^ mice. (**a**) Representative *Mlc1*-ErbB2^V664E^ and littermate control mice at P40 with Dox withdrawal from P21. Note *Mlc1*-ErbB2^V664E^ mice were much smaller than their littermate controls. (**b**) Quantitative analysis of the weight of *Mlc1*- ErbB2^V664E^ and littermate control mice at P40 with Dox withdrawal from P21. **P=0.0018, *n*=4 for each group, paired* t-*test. (**c**) Isolated digestive systems from *Mlc1*-ErbB2^V664E^ and littermate control mice at P40 with Dox withdrawal from P21. Note the atrophic gastrointestinal tract and accessory organs of *Mlc1*-ErbB2^V664E^ mice because of malnourishment during adolescent development. (**d**) No pathological changes in digestive system of *Mlc1*-ErbB2^V664E^ mice at P40 with Dox withdrawal from P21. H & E staining results showed that stomach walls of *Mlc1*-ErbB2^V664E^ mice folded in on itself because of no content in gastrointestinal tract. (**e**) Reactive astrogliosis and associated inflammation in the hypothalami of *Mlc1*-ErbB2^V664E^ mice as indicated by increased immunoreactivities of GFAP and Iba1, respectively. (**f**) Brain appearance and gross structure showed no apparent difference except enlarged ventricles in *Mlc1*-ErbB2^V664E^ mice. Shown are the whole brain and H & E stained brain slices of *Mlc1*-ErbB2^V664E^ and littermate control mice at P40 with Dox withdrawal from P21.

Compared with their littermate controls, *Mlc1*-ErbB2^V664E^ mice consumed much less food and water after Dox withdrawal. Twenty days after Dox withdrawal, they exhibited malnourishment with significantly smaller body sizes and lighter weights (Figure 5a and b). To explore whether there was a peripheral problem causing anorexia, we dissected their digestive systems and discovered atrophy of the gastrointestinal tract and accessory organs (Figure 5c). Further investigation of gastrointestinal tract sections with hematoxylin and eosin (H & E) staining showed no pathological changes, although the stomachs were constricted because of lack of content (Figure 5d). There were no cells targeted by *Mlc1*-tTA in the gastrointestinal tract of *Mlc1*-ErbB2^V664E^ mice (Supplementary Figure S11a and b). Therefore, anorexia was caused by an inhibition of neural circuits for feeding behavior. Remarkably, hypothalamic astrocytes have crucial roles in controlling feeding behavior [44], and hypothalamic inflammation is linked to anorexia [45]. Indeed, astrocytic ErbB activation induced prevalent astrogliosis and associated inflammation in the brain including the hypothalamus, as indicated by intensively stained GFAP^+^ and Iba1^+^ cells there (Figure 5e). To screen for other possible pathological changes, mouse brains were sectioned for H & E staining. Astrocyte dysfunction and associated inflammation would disrupt the integrity of the brain–blood barrier and influence cerebrospinal fluid production [46]. As a result, the ventricles of *Mlc1*-ErbB2^V664E^ mice were larger than those of littermate controls. Nevertheless, other brain regions exhibited comparable sizes in the two groups (Figure 5f). Therefore, in animals with reactive astrogliosis and associated inflammation throughout the brain, anorexia was the predominant result, suggesting hypothalamic susceptibility.

### ErbB activation stimulated multiple downstream signaling pathways in reactive astrocytes

**Figure 6:**
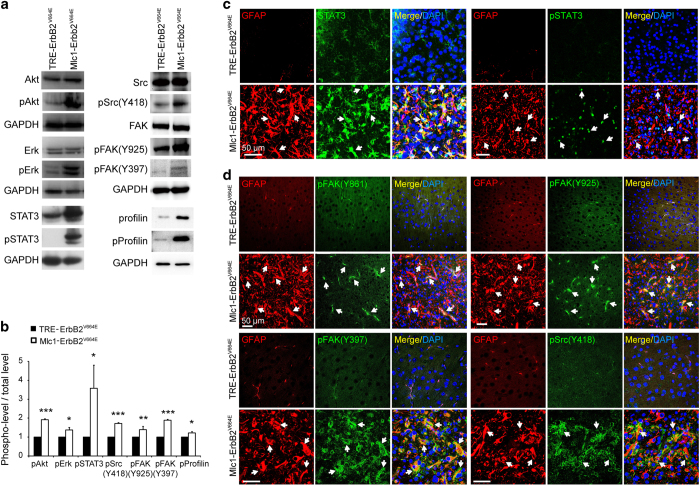
Molecular signaling activated in reactive astrocytes induced by cell-autonomous ErbB activation. (**a**) Various intracellular signaling proteins were activated in the cerebral cortices of *Mlc1*-ErbB2^V664E^ mice, as increases of their phosphorylation levels were indicated by WB results. (**b**) Quantitative analyses of the protein phosphorylation levels revealed by WB. Phosphorylation levels of indicated proteins were normalized by its total protein levels. ***P<0.001; **P<0.01; *P<0.05; *n*=3 for each protein, paired* t-*test. (**c**) Specific elevation of both total protein levels and activity of STAT3 in the reactive astrocytes of *Mlc1*-ErbB2^V664E^ mice. Cortical slices of *Mlc1*-ErbB2^V664E^ and littermate control mice were co-immunostained for STAT3 or phosphorylated STAT3 (pSTAT3) together with GFAP. White arrows, representative double positive cells. (**d**) Specific activation of FAK and Src in the reactive astrocytes of *Mlc1*-ErbB2^V664E^ mice. Cortical slices of *Mlc1*-ErbB2^V664E^ and littermate control mice were co-immunostained by mouse antibody against GFAP and rabbit/goat antibodies against the active forms of FAK or Src, respectively. White arrows, representative double positive cells. Note that one of the active form of FAK (pY397) exhibited similar subcellular distribution pattern to the active form of Src (pY418).

Next, we investigated which downstream signaling pathways were activated by cell-autonomous activation of ErbB signaling in astrocytes. Because cultured astrocytes differ from *in vivo* astrocytes [25, 47], we first examined various candidate signaling proteins in brain tissues. Based on WB assays, Akt and Erk, the classic ErbB downstream signaling proteins that are important for astrogliosis, were indeed active in *Mlc1*-ErbB2^V664E^ cortices (Figure 6a and b). In addition, the classic inflammatory signaling protein, STAT3, which is critical for astrogliosis [10, 13, 48], markedly increased in both level and activity in cortical tissues from *Mlc1*-ErbB2^V664E^ mice (Figure 6a and b). Moreover, our immunostaining results revealed specific activation and increased levels of STAT3 protein in reactive astrocytes in *Mlc1*-ErbB2^V664E^ mice (Figure 6c).

**Figure 7:**
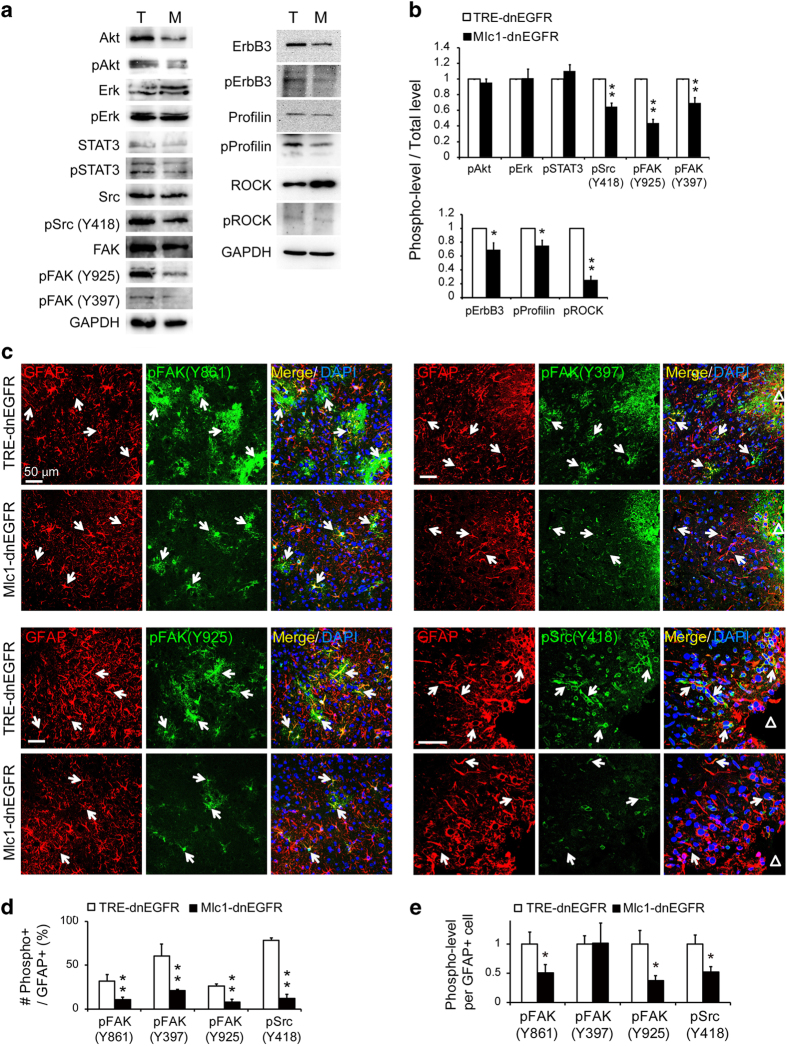
Molecular signaling in the regulation of reactive astrocyte hypertrophy. (**a**) Activities of various signaling proteins in injured cortices of *Mlc1*-dnEGFR (M) and littermate control mice (T) 3 days post injury were examined by WB. Note the specific reduction of phosphorylated FAK (pFAK) and Src (pSrc), as well as profilin (pProfilin), in the injured cortical tissues from *Mlc1*-dnEGFR mice in comparison with that from littermate controls. (**b**) Quantitative analyses of the protein phosphorylation levels revealed by WB. Phosphorylation levels of indicated proteins were normalized by its total protein levels. **P<0.01; *P<0.05; *n*=3 for each protein, paired* t-*test. (**c**) Specific reduction of FAK and Src activities in the reactive astrocytes of *Mlc1*-dnEGFR mice in comparison with that of littermate controls. Cortical slices from injured *Mlc1*-dnEGFR and littermate control mice were co-immunostained by mouse antibody against GFAP and rabbit/goat antibodies against the active forms of FAK or Src, respectively. White arrows, representative double positive cells. ‘Δ’ represents the injury sites. (**d**) Percentage of reactive astrocytes with active FAK or Src according to immunostaining results. Only cells positive for GFAP were analyzed. **P<0.01; *P<0.05; *n*=4 for each group, paired* t-*test. (**e**) Average levels of FAK or Src phosphorylation in individual reactive astrocytes according to immunostaining results. Only cells positive for GFAP were analyzed. *P<0.05; *n*=4 for each group, paired* t-*test.

In addition, WB revealed that Src and FAK activity were significantly increased in the cortices of *Mlc1*-ErbB2^V664E^ mice (Figure 6a and b). Moreover, these two non-receptor tyrosine kinases were specifically activated in reactive astrocytes, as indicated by the immunoreactivities of their multiple active forms being localized in GFAP^+^ cells (Figure 6d). Both Src and FAK are downstream of ErbB receptors [49, 50], and participate in regulating multiple brain functions [51, 52]. Note that the subcellular distribution pattern of FAK with phosphorylation at Y397 in reactive astrocytes was similar to that of the active form of Src (pY418) (Figure 6d), consistent with a previous report that FAK interacts with Src through phosphorylated Y397 [53]. Src and FAK both mediate signaling pathways that regulate actin polymerization [49, 54]. Consistent with this idea, both protein and phosphorylation levels of profilin, an actin-binding protein that regulates actin polymerization and astrocytic morphology [55], was increased in the cortices of *Mlc1*-ErbB2^V664E^ mice (Figure 7a and b).

### Differentiated regulation of multifaceted reactivity in astrocytes by ErbB signaling

Although cell-autonomous activation of ErbB signaling in mature astrocytes induced spontaneous astrogliosis with canonical features (Figure 4), inhibiting ErbB signaling in reactive astrocytes specifically suppressed their ability to develop hypertrophy (Figure 2). To investigate which signaling pathways mediated the effects of ErbB activation on the hypertrophy of reactive astrocytes, ErbB-activated downstream signaling proteins were examined in the injured cortices of *Mlc1*-dnEGFR and littermate control mice. Interestingly, WB revealed no reductions in phosphorylated Akt, Erk or STAT3, despite the fact that ErbB3 activity was reduced in injured tissues of *Mlc1*-dnEGFR mice (Figure 7a and b). Instead, phosphorylation of FAK and Src was significantly suppressed in injured tissues from *Mlc1*-dnEGFR mice (Figure 7a and b). Further, immunostaining revealed that stab wound injury in control brains induced upregulation of the active forms of FAK (pY861, pY397 and pY925) and Src (pY418) specifically in reactive astrocytes (Figure 7c and Supplementary Figure S12). However, both the number of cells with active FAK or Src and the average activity per cell were significantly reduced in the injured cortices of *Mlc1*-dnEGFR mice (Figure 7c–e). In concordance with the dnEGFR-induced suppression of reactive astrocytic expansion, phosphorylation of profilin, as well as its upstream regulating kinase ROCK [56], were suppressed in the injured cortices of *Mlc1*-dnEGFR mice (Figure 7a and b). Consistent with the finding that hypertrophic remodeling did not rely on GFAP expression (Figure 2), the discovery of a Src/FAK/profilin signaling pathway emphasized the involvement of active actin remodeling in the reactive responses of astrocytes.

**Figure 8:**
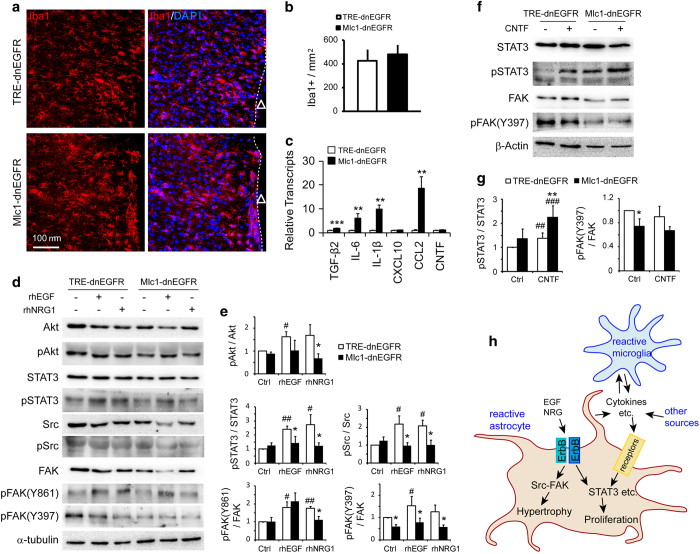
Inhibition of ErbB signaling in reactive astrocytes did not block inflammatory signaling induced by injury. (**a**) Similar inflammation induced by stab injury in the cortices of *Mlc1*-dnEGFR and littermate control mice. Shown are representative images of Iba1 immunostaining in injured cortical regions 3 days post injury. ‘Δ’ represents the injury sites. (**b**) Quantitative analysis of Iba1^+^ cell densities in injured cortical regions of *Mlc1*-dnEGFR and littermate control mice. *n*=3 for each group, paired* t-*test. (**c**) Real-time RT-PCR results of indicated cytokines in injured cortical tissues from *Mlc1*-dnEGFR and littermate control mice 7 days post injury. ***P<0.001; **P<0.01; *n*=3 for each group. (**d**) Activities of various signaling proteins induced by ErbB receptor ligands were reduced in primary *Mlc1*-dnEGFR astrocytes. Primary astrocytes from *Mlc1*-dnEGFR and control mice were treated with saline (Ctrl), rhEGF (1 μg/ml), or rhNRG1 (100 ng/ml) for 15 min, respectively, and indicated proteins and their phosphorylation levels in cell lysates were examined by WB. (**e**) Quantitative analyses of the protein phosphorylation levels revealed by WB in astrocytes stimulated by ErbB ligands. Phosphorylation levels of indicated proteins were normalized by its total protein levels. ^#^*P*<0.05; ^##^*P*<0.01; as compared with the treatment with saline. *P<0.05, as compared with control astrocytes with the same treatment. *n*=3 for each protein, paired* t-*test. (**f**) STAT3 activity induced by cytokine CNTF was not reduced in primary *Mlc1*-dnEGFR astrocytes. Primary astrocytes from *Mlc1*-dnEGFR or control mice were treated with saline (Ctrl) or CNTF (200 ng/ml) for 30 min and indicated proteins and their phosphorylation levels in cell lysates were examined by WB. (**g**) Quantitative analyses of the protein phosphorylation levels revealed by WB in astrocytes stimulated by CNTF. Phosphorylation levels of indicated proteins were normalized by its total protein levels. ^##^*P*<0.01; ^###^*P*<0.001; as compared with the treatment with saline. **P<0.01, as compared with control astrocytes with the same treatment. *n*=3 for each protein, paired* t-*test. (**h**) Schematic illustration of a working model for the role of ErbB signaling in the induction of reactive astrogliosis. ErbB activation in quiescent astrocytes initiates reactive astrogliosis via diverse downstream signaling pathways. Src and FAK, the non-receptor tyrosine kinases regulating actin polymerization, are activated by ErbB signaling to prompt hypertrophic remodeling in astrocytes. Other signaling proteins downstream of ErbB receptors, such as STAT3, could be activated through different pathways stimulated by multiple factors in the inflammatory environment. Inhibiting ErbB signaling in reactive astrocytes blocks hypertrophy through a direct inhibition on Src/FAK activities, whereas other signaling proteins such as STAT3 remain active to promote proliferation. Note that microglia could react to factors released from reactive astrocytes or other sources.

Given the clear phosphorylation of STAT3 in the astrocytes of *Mlc1*-ErbB2^V664E^ mice after Dox withdrawal (Figure 6a–c), we were surprised to see comparable STAT3 phosphorylation (pSTAT3) levels between injured cortical tissues from *Mlc1*-dnEGFR and littermate control mice (Figure 7a and b). However, as it is a typical inflammatory signal, STAT3 can be activated by various cytokines in addition to ErbB receptors [57]. To ensure that other activators of the STAT3 pathway were unaffected in injured *Mlc1*-dnEGFR brains, we examined the inflammatory status of the injured cortices by Iba1 immunostaining to label reactive microglia and by real-time RT-PCR to assess the levels of the following cytokines: CXCL10, CCL2, IL-1β, IL-6, CNTF and TGF-β2. We found no difference in Iba1^+^ cell densities between *Mlc1*-dnEGFR and littermate mice in cortical regions adjacent to the sites of injury (Figure 8a and b). Moreover, with the exception of CXCL10 and CNTF, which showed no change, mRNA levels of CCL2, IL-1β, IL-6 and TGF-β2 were significantly increased in injured tissues from *Mlc1*-dnEGFR mice (Figure 8c). These results indicated that ErbB inhibition in reactive astrocytes did not suppress local inflammation induced by brain injury. Moreover, many studies have revealed that reduced astrocyte reactivity aggravates local inflammation [10, 13, 58–60]. Therefore, the observed increase in cytokine transcription in injured *Mlc1*-dnEGFR cortices may indirectly reflect attenuated astrocyte reactivity induced by cell-autonomous inhibition of ErbB signaling.

In summary, our findings revealed differentiated regulation of aspects of astrocyte reactivity by ErbB activation. Reactive astrocytes were situated in a niche comprising autonomously released pathological stimuli from various cell types. The non-altered signaling pathways in injured *Mlc1*-dnEGFR cortices, such as STAT3, were likely affected by multiple upstream regulators. In contrast, Src and FAK were directly regulated by ErbB signaling in reactive astrocytes for hypertrophic regulation (Figure 8h). In supporting our hypothesis, activities of downstream signaling stimulated by rhEGF or rhNRG1, including Akt, STAT3, FAK or Src, were compromised in primary *Mlc1*-dnEGFR astrocytes in comparison with that in control astrocytes (Figure 8d and e). In contrast, STAT3 activity induced by cytokine CNTF was not reduced in primary *Mlc1*-dnEGFR astrocytes. Instead, it was more increased by CNTF in primary *Mlc1*-dnEGFR astrocytes than that in control cells, in line with the *in vivo* observation that inflammatory status was aggravated in the injured cortices of *Mlc1*-dnEGFR mice (Figure 8f and g). The dissociated regulation of aspects of astrocyte reactivity indicated a multifaceted implication of reactive astrocytes in diseased or injured brains.

## Discussion

Reactive astrocytes are being considered as therapeutic targets for various neurological disorders [4, 39, 58]. Here, we showed the role of ErbB signaling as a positive upstream regulator in reactive astrogliosis. Different from overexpressing a secreted ligand [61], we targeted receptors, which specifically regulated the signaling in astrocytes *in vivo* by using mice genetically engineered for inducible gene expression. We showed that ErbB activation was sufficient to mediate the reactive responses of mature astrocytes, prompting molecular and morphological changes characteristic of injury and disease-induced astrogliosis (Figure 4). We also revealed that many intracellular signals critical for astrogliosis, including STAT3, were stimulated by ErbB activation in astrocytes (Figures 6 and 8). This is in marked contrast to FGF signaling that inhibits astrocyte reactivity [18], in spite of both ErbB receptors and FGFRs being able to activate similar sets of downstream signaling proteins [15, 16].

Interestingly, the inhibition of brain injury-induced ErbB signaling in reactive astrocytes suppressed hypertrophic remodeling without affecting proliferation (Figures 1, 2, 3). Further, Src/FAK activities were specifically blocked by inhibiting ErbB signaling in reactive astrocytes (Figure 7). Src and FAK are frequently connected to the regulation of cellular processes [53, 62, 63], and most of their effects occur through regulating actin remodeling [49, 50, 54, 64]. Given that hypertrophy could be established independently of GFAP in cortical reactive astrocytes (Figure 2g), we postulated that it was most likely caused by actin remodeling. Consistent with this hypothesis, we determined that phosphorylated profilin, which regulates actin polymerization, was reduced following ErbB inhibition in reactive astrocytes (Figure 7a and b). GFAP expression was also suppressed in injured cortices of *Mlc1*-dnEGFR mice (Figure 2d and e), which helped limit cellular process extension [31]. Together, these results suggested that ErbB signaling positively regulated astrocyte reactivity, with prominent and direct effects on hypertrophic remodeling. Being a well-known sign of reactive responses, hypertrophy might actively participate in cell–cell contact, endocytosis, phagocytosis and migration by the mediation of actin remodeling. In-depth work is worth pursuing to define the pathological contribution of hypertrophy or other actin-involved cellular activities of glial cells in reactive astrogliosis.

There have been several reports suggesting that morphological changes and proliferation are dissociated in reactive astrocytes. First, injury-induced astrogliosis in GFAP/vimentin-deficient mice exhibits astrocyte proliferation comparable to that in wild-type mice [65]. Moreover, spontaneous astrogliosis in mice deficient in FGFRs does not result in proliferation of reactive astrocytes [18]. Immunogenic capacity is also likely dissociated from the proliferation and hypertrophy in reactive astrocytes, as spontaneous astrogliosis in mice deficient in Bax does not recruit reactive microglia [66]. Nevertheless, the dissociated regulation of aspects of astrocyte reactivity is not clearly understood. The discrepancies between results obtained from *Mlc1*-ErbB2^V664E^ and *Mlc1*-dnEGFR mice indicated that the proliferation of reactive astrocytes was regulated by signaling downstream of the initial reactive responses induced by ErbB activation and that it could be independent of ErbB receptor activation in astrocytes.

Many molecular signals released from various cell types are involved in regulating astrocyte proliferation after CNS injury. For example, FGF, Sonic hedgehog, endothelin-1 and STAT3 signaling pathways promote the proliferation of reactive astrocytes [18, 67–69]. Intriguingly, inhibiting inflammation abolishes both Sonic hedgehog activation and proliferation of reactive astrocytes in injured brains [35]. STAT3 can be activated by FGFRs, ErbB receptors and cytokine receptors [57, 70, 71]. Note that reactive astrogliosis is usually complicated by interactions among reactive astrocytes, microglia, endothelial cells and other cell types [39]. We found that although reactive astrocytes caused by cell-autonomous ErbB activation induced local inflammation (Supplementary Figure S9), immunogenic stimuli released from non-astrocytes could not be blocked by ErbB inhibition in astrocytes. In fact, the number of reactive microglia (Iba1^+^ cells) and cytokine mRNA levels were not reduced in injured cortices of *Mlc1*-dnEGFR mice (Figure 8a–c). Moreover, STAT3 activity (pSTAT3) was not reduced in either injured cortical tissues or CNTF-treated primary astrocytes from *Mlc1*-dnEGFR mice (Figures 7 and 8). These results suggest that some aspects of astrogliosis, including proliferation, are regulated by many coordinated signaling pathways that are stimulated by reactive astrocytes themselves or by other sources (Figure 8h, working model). Among them, the mechanism by which ErbB receptors and FGFRs antagonize each other in reactive astrocytes and cross-talk with STAT3 pathways through inflammatory signals deserves further investigation.

No matter how heterogeneous astrocytes in different brain regions are [25], cell-autonomous ErbB activation in astrocytes induced similar reactive responses throughout the brain (Figure 4 and Supplementary Figures S7–S9). Intriguingly, prevalent astrogliosis and associated brain inflammation predominantly caused anorexia in animals (Figure 5). The susceptibility of different brain regions to reactive astrogliosis warrants further investigation. Nevertheless, the functional roles of astrogliosis in pathological development are still debated. For example, glial scar formation over injury sites has been shown to restrain damage but perturb axon regrowth in the CNS [72, 73]. However, a recent study reported astrocytic scars to be beneficial for axon regeneration [74]. This paradox was solved by a more recent work that disrupting the molecular signaling specifically transforming reactive astrocytes into scar-forming astrocytes leads to enhanced axon regrowth, indicating a functional dissociation of different types of astrocytes that are sequentially induced during the development of reactive astrogliosis [75]. Moreover, the type of CNS insult influences the outcome of astrogliosis in a context-dependent manner [5, 7]. Therefore, signaling pathways regulating astrocyte reactivity must be assessed in specific contexts to comprehensively evaluate the targets that contribute to or inhibit astrogliosis. It would be intriguing to determine whether ErbB inhibition alters functions other than scar formation in reactive astrocytes and whether these functions are required for specific pathological processes. Our work shed light on the molecular mechanisms that regulate astrocyte reactivity and described inducible animal models that could be useful for future investigations into the function of astrogliosis and potential therapeutic targets.

## Materials and methods

### Animals

*Mlc1*-tTA transgenic mice were from the RIKEN Bioresource Center (stock no. RBRC05450). Transgenic mice *TRE*-ErbB2^V664E^ (stock no. 010577) and *TRE*-dnEGFR (stock no. 010575) were from the Jackson Laboratory. *Mlc1*-tTA;*TRE*-ErbB2^V664E^ (*Mlc1*-ErbB2^V664E^) and *Mlc1*-tTA;*TRE*-dnEGFR (*Mlc1*-dnEGFR) were obtained by breeding *Mlc1*-tTA mice with *TRE*-ErbB2^V664E^ or *TRE*-dnEFGR mice, respectively. Primers Mlc1U-657 (5′- AAATTCAGGAAGCTGTGTGCCTGC-3′) and mtTA24L (5′- CGGAGTTGATCACCTTGGACTTGT-3′) with a 680- bp PCR product were used for genotyping of *Mlc1*-tTA, whereas primers 9707 (5′- AGCAGAGCTCGTTTAGTG-3′) and 9708 (5′- GGAGGCGGCGACATTGTC-3′) with a 625- bp PCR product for that of *TRE*-ErbB2^V664E^, and primers 9013 (5′- TGCCTTGGCAGACTTTCTTT-3′) and 7554 (5′- ATCCACGCTGTTTTGACCTC-3′) with a 318-bp PCR products for that of *TRE*-dnEGFR. Unless indicated, mice were housed in a room with a 12-h light/dark cycle with access to food and water *ad libitum*. *Mlc1*-dnEGFR, *Mlc1*-tTA and *Mlc1*-ErbB2^V664E^ mice with either sex and their littermate control mice with matched sex were used for experiments. Animal experiments were approved by the Institutional Animal Care and Use Committee of the Hangzhou Normal University.

### Tet-off treatment of mice

The pregnant mice and their offspring were fed with tetracycline analog, Dox (0.5 mg/ml, 10 ml/day), in drinking water to inhibit the expression of ErbB2^V664E^ in *Mlc1*-ErbB2^V664E^ mice from embryonic to indicated postnatal days. Water bottles were wrapped with foil to protect Dox from light. *Mlc1*-dnEGFR mice and their littermate controls were not treated with Dox for they did not exhibit developmental difference. Withdrawal from Dox induces the expression of ErbB2^V664E^ and dnEGFR in astrocytes of *Mlc1*-ErbB2^V664E^ and *Mlc1*-dnEGFR mice. All used littermate control mice were treated the same.

### Antibodies and reagents

Information on commercial rabbit antibodies is as follows: EGFR (1902-1, 1:5000 for WB) and pEGFR (Tyr1068, 1727-1, 1:2500 for WB) were from Epitomics (Burlingame, CA, USA); ErbB2 (sc-284, 1:1000 for WB, 1:200 for immunofluorescence (IF)), ErbB3 (sc-285, 1:5000 for WB, 1:50 for IF), ErbB4 (sc-283, 1:5000 for WB) and pFAK (Tyr397, sc-11765-R, 1:200 for WB, 1:50 for IF) were from Santa Cruz (Dallas, TX, USA); ROCK2 (9029, 1:1000 for WB), Erk1/2 (9102, 1:5000 for WB), pErk1/2 (Thr202/Tyr204, 4370, 1:5000 for WB), Akt (9272, 1:5000 for WB), pAkt (Ser473, 9271, 1:3000 for WB) and pSTAT3 (Tyr705, 9145, 1:2000 for WB, 1:100 for IF) were from Cell Signaling (Danvers, MA, USA); Ki67 (RB-9043-P1, 1:500 for IF) was from Thermo (Fremont, CA, USA); Glyceraldehyde-3-phosphate dehydrogenase (ABS16, 1:2500 for WB), GFAP (AB5804, 1:2000 for IF) and Olig2 (AB9610, 1:500 for IF) were from Millipore (Temecula, CA, USA); pROCK2 (ab182648, 1:500 for WB), pSrc (Tyr418, ab4816, 1:1000 for WB, 1:100 for IF), Acsbg1 (ab65154, 1:500 for IF), pErbB3 (Tyr1328, ab133459, 1:2500 for WB, 1:100 for IF) and pErbB4 (Tyr1284, ab109273, 1:2500 for WB) were from Abcam (Cambridge, MA, USA); pProfilin (Tyr129, PP4751, 1:1000 for WB) was from ECM Biosciences (Versailles, KY, USA); AQP4 (HPA014784, 1:2500 for IF) and GS (G2781, 1:200 for IF) were from Sigma (St Louis, MO, USA). Information on commercial mouse antibodies is as follows: pErbB2 (Tyr1140, AP3781q, 1:2500 for WB) was from Abgent (San Diego, CA, USA); STAT3 (9139,1:1000 for WB, 1:1600 for IF)) was from Cell Signaling; MBP (MAB382, 1:1000 for WB), GFAP (MAB360, 1:3000 for WB, 1:2000 for IF) and Iba1 (MABN92, 1:1000 for IF) were from Millipore; Nestin (ab11306, 1:100 for IF) and Aldh1L1 (ab56777, 1:200 for IF) was from Abcam; RC2 (1:100 for IF) was from Developmental Studies Hybridoma Bank (Iowa city, IA, USA). Information on commercial goat antibodies is as follows: pFAK (Tyr861, sc-16663, 1:200 for WB, 1:50 for IF) and pFAK (Tyr925, sc-11766, 1:200 for WB, 1:50 for IF) were from Santa Cruz. Rat antibodies to CD45 (103101, 1:200 for IF) was from BioLegend (San Diego, CA, USA). Alexa Fluor 488- and Alexa Fluor 594-conjugated secondary antibodies (1:1000 for IF) were purchased from Invitrogen (Rockford, IL, USA), and horseradish peroxidase-conjugated secondary antibodies (1:5000 for WB) were from CWBIO (Beijing, China). Recombinant rat CNTF (cat. no. 557-NT-010/CF), rhEGF (cat. no. 236-EG-200) and recombinant human NRG1 (cat. no. 377-HB-050/CF) were from R&D (Minneapolis, MN, USA). Dox and TRIzol were from Sangon (Shanghai, China). Fluorescent mounting medium was from CWBIO. Protease inhibitor cocktail and bioinchoninic acid assay kit were from Thermo. EZ-ECL was from Biological Industries (Cromwell, CT, USA). PrimeScript Reverse Transcriptase was from Takara (Shiga, Japan). SYBR Green PCR mixture was from Bio-Rad (Hercules, CA, USA). All other chemicals were from Sigma.

### Stab wound injury

Cortical stab injuries were operated under a stereotaxic apparatus (RWD68025) on mice at age of P30. Mice were anesthetized by 1% pentobarbital (50 mg/kg, i.p.). The parietal skull was exposed and made a small hole by a micro-drill. Traumatic brain injury was made by stabbing with a needle (0.5 mm in diameter) in the cortex at 1.5 mm lateral to the midline, 2 mm posterior to bregma and 1 mm deep from the surface of meninges. Different days after the surgery, brains were isolated and fixed with 4% paraformaldehyde (PFA) in 0.1 M phosphate buffer (PB) and sectioned by vibrating microtome. Astrogliosis was verified by immunostaining for GFAP. Image J (NIH, Bethesda, MD, USA) was used to analyze and quantify the size and number of GFAP^+^ cells, as well as scar thickness, in consecutive sections over the injury sites. To study the scar formation, mouse cortices were stabbed by a scalpel blade (#11, angled with 20-mm cutting edge and 0.4-mm thickness, Shanghai Chengyuan Medical Supplies Factory, Shanghai, China) parallel to the longitudinal fissure at 1.5 mm lateral to the midline, 2 mm posterior to bregma and 1 mm deep from the surface of meninges. For WB and real-time RT-PCR, injured sites were identified through the drilled holes in the skull and injured cortical tissues were isolated from uninjured part. We usually combined the injured cortical tissues from several mice with the same genotype to serve one sample for each batch of experiment.

### Stereotaxic injection of AAV viruses

AAV-*TRE*-YFP plasmids were constructed by standard methods, packaged as AAV9 viruses, and produced with titers of 1×10^12^ particles per ml by OBio (Shanghai, China). Mice were anesthetized by 1% pentobarbital (50 mg/kg, i.p.) and mounted at stereotaxic apparatus (RWD68025). AAV-*TRE*-YFP (1 μl) was injected into the cortex (from bregma in mm, cortex, M-L: ±0.9, A-P: −1.0, D-V: 1.0) under the control of micropump (KDS310) at speed of 0.07 μl/min. Injecting needles (Hamilton NDL ga33/30 mm/pst4, Switzerland) were withdrawn 5 min after injection. To observe the cell specificity of *Mlc1*-tTA, injected brains were isolated and fixed in 4% PFA in PB 1 day after injection to avoid reactive gliosis induced by virus injection. To analyze the sizes of reactive astrocytes, viruses were injected near the injury sites 1.5, 3 or 7 days post injury, and injected brains were isolated and fixed 1 day later. Fixed brains were sectioned and immunostained for GFAP or Acsbg1, and images were taken by a Zeiss LSM710 confocal microscope (Berlin, Germany). Morphology of cells was observed by maximum projection of Z-stacked images taken under a 40X oil-immersion objectives. When analyzing the Z-stacked images, it was shown that the astrocytic areas around cell bodies within 6-μm Z-axial range were the biggest (Supplementary Figure S5b). For the quantitative purpose as shown in Figure 2, big fields were captured under a 20X objective, and the pinhole was set at 200 in order to acquire YFP^+^ cells with most of cell bodies and their processes in optical slices with 6.1-μm thickness. Captured cells with complete cell bodies were subjected to size measurement. YFP fluorescence was imaged with exactly same scanning conditions for paired experiments, and sizes of YFP-labeled cells were measured by Image J.

### Dye-filling of astrocytes

AAV-*TRE*-YFP-labeled cells in fixed brain slices were filled with dye by methods described previously [31, 32]. Briefly, mice with injured cortices 1 day after stereotaxic injection of AAV-*TRE*-YFP were anesthetized by 1% pentobarbital and transcardially perfused with oxygenated Ringer’s solution (1.35 mM NaCl, 0.05 mM KCl, 0.01 mM MgCl_2_·6H_2_O, 0.013 mM Na_2_HPO_4_, 0.15 mM NaHCO_3_, 0.02 mM CaCl_2_·2H_2_O, 0.11 mM dextrose, 0.0085 mM xylocaine) and then with 4% PFA in phosphate-buffered saline (PBS; pH 7.4). Isolated brains were postfixed in 4% PFA in PBS for 1 h, and then sectioned into 75-μm slices by vibrating microtome. YFP-labeled astrocytes in the cerebral cortex were identified under a fluorescence microscope, and were patched and impaled with glass micropipettes (o.d., 1.00 mm; i.d., 0.58 mm; resistance, 100–400 MΩ) that had been backfilled with 10 mM Alexa Fluor 568 (Invitrogen) in 200 mM KCl. Patched astrocytes were iontophoretically injected with the dye by using 1-s pulses of negative current (0.5 Hz) for 2 min. After several cells were filled, the slices were placed in ice-cold 4% PFA overnight and then mounted under coverslips. Dye filled astrocytes were observed under a Zeiss710 confocal microscope equipped with a 40X oil-immersion objective, and Z-stack images were captured and projected to evaluate the subcellular distribution of injected dye and YFP.

### IF staining

Mouse brains were isolated and fixed in 4% PFA in 0.1 M PB (0.019 M NaH_2_PO_4_, 0.089 M Na_2_HPO_4_, pH 7.4) overnight, and then washed with 0.1 M PB twice. The fixed brains were kept in 0.1 M PB with 1% ProClin 200 in 4 °C until sectioned by vibrating microtome. Soft agar-embedded mouse brains were cut into 50 μm sections and subjected to immunostaining as previously described [76]. Briefly, brain slices were incubated with blocking buffer (10% fetal bovine serum and 0.1% Triton-X-100 in 0.1 M PB) for 1 h at room temperature, and then incubated at 4 °C overnight with primary antibodies diluted in blocking buffer. After washing three times with PB, samples were incubated at room temperature for 1 h with Alexa-488 or −594 secondary antibody, and then washed and mounted on adhesion microscope slides (CITOTEST) with fluorescent mounting medium. For co-immunostaining, samples were incubated with the second primary antibody the next morning for 1 h at room temperature before staining with the secondary fluorescence antibodies. Images were taken by a Zeiss LSM710 confocal microscope with exactly same scanning conditions for paired experiments, and analyzed by Image J.

### Western blotting

Different brain regions were isolated and homogenized. For injured brains, only the tissues surrounding the injury sites were collected and homogenized. Homogenates in lysis buffer (10 mM Tris-Cl, pH 7.4, 1% NP-40, 0.5% Triton-X 100, 0.2% sodium deoxycholate, 150 mM NaCl, 20% glycerol, protease inhibitor cocktail) at ratio of 1 ml per 100 mg tissue were lysed overnight in 4 °C. Lysates were centrifuged at 12 000 *g* and 4 °C for 30 min to get rid of the unsolved debris. Concentration of the supernatant was measured by BCA assay. Proteins in samples were separated by 6–12% sodium dodecyl sulfate–polyacrylamide gel electrophoresis, transferred to a polyvinylidene fluoride membrane (Millipore), and then incubated with indicated primary antibodies at 4 °C overnight after blocking by 5% non-fat milk solution in Tris-buffered saline with Tween-20 (50 mM Tris, pH 7.6, 150 mM NaCl, 0.1% Tween 20) for 1 h at room temperature. Next day, the membranes were washed by Tris-buffered saline with Tween-20 for three times and incubated with the secondary antibodies for 1 h at room temperature. Membranes were washed again and incubated with substrate EZ-ECL for visualization of chemiluminescence by exposure to X-ray films or Bio-Rad GelDOCXR^+^ Imaging System. Intensities of protein bands were measured by Image J (NIH), and statistical analysis was performed after subtraction of the background intensity.

### Histological examination

Fixed tissues were kept in 0.1 M PB with 1% ProClin 200 in 4 °C until sectioned by vibrating microtome. Soft agar-embedded mouse brains were cut into 30 μm sections and subjected to H & E staining. In brief, brain slices were stained with hematoxylin solution for 15 min and then rinsed in running tap water for 10 min before staining with eosin solution for 5 min. After rinsing with running tap water for 10 min, stained slices were mounted on adhesion microscope slides with resin-based mounting medium. Phase contrasted images were taken by a Zeiss Observer A1 inverted microscope under bright field with transmitted-light.

### Real-time RT-PCR

Total RNA was extracted from isolated mouse brains using TRIzol following the manufacturer’s protocol. Complementary DNA was synthesized by using the PrimeScript Reverse Transcriptase. Real-time PCR was performed in four repeats for each sample by using SYBR Green PCR mixture with the Bio-Rad CFX96 real-time PCR system as previously described [76]. Relative mRNA levels were analyzed by Bio-Rad CFX Manager. Transcripts of targeted genes were normalized to these of mouse 18s ribosomal RNA gene in the same samples. Primers for 18s ribosomal RNA were 5′- CGGACACGGACAGGATTGACA-3′ and 5′- CCAGACAAATCGCTCCACCAACTA-3′ with a 94-bp PCR product. Primers for mouse gene *EGFR* and transgene *dnEGFR* were 5′- TCCTGCCAGAATGTGAGCAG-3′ and 5′- ACGAGCTCTCTCTCTTGAAG-3′ with a 500-bp PCR product. Primers for mouse gene *IL-6* were 5′- GGGACTGATGCTGGTGACAACC-3′ and 5′- CATGTGTAATTAAGCCTCCGACTTGTG-3′ with a 128- bp PCR product. Primers for mouse gene *IL-1Î²* were 5′- GGCAGGCAGTATCACTCATTGTG-3′ and 5′- TGTCCTCATCCTGGAAGGTCC-3′ with an 84- bp PCR product. Primers for mouse gene *CCL2* were 5′- TCACCTGCTGCTACTCATTC-3′ and 5′- GTAGGTTCTGATCTCATTTGGTTCC-3′ with a 205-bp PCR product. Primers for mouse gene *CXCL10* were 5′- GTCTGAGTGGGACTCAAGGGATCCC-3′ and 5′- CATCGTGGCAATGATCTCAACACGT-3′ with a 155- bp PCR product. Primers for mouse gene *TGF-Î²2* were 5′- TGCTTCGAATCTGGTGAAGGCA-3′ and 5′- GGAGAGCCATTCACCCTCCGCT-3′ with a 181- bp PCR product. Primers for mouse gene *CNTF* were 5′- TTTCGCAGAGCAATCACC-3′ and 5′- AATTGTGACAGGCATCC-3′ with a 433-bp PCR product. Primers for mouse gene *H2-D1* were 5′-TCCGAGATTGTAAAGCGTGAAGA-3′ and 5′- ACAGGGCAGTGCAGGGATAG-3′ with a 204-bp PCR product. Primers for mouse gene *Serping 1* were 5′- ACAGCCCCCTCTGAATTCTT-3′ and 5′- GGATGCTCTCCAAGTTGCTC-3′ with a 299-bp PCR product. Primers for mouse gene *H2-T23* were 5′- GGACCGCGAATGACATAGC-3′ and 5′- GCACCTCAGGGTGACTTCAT-3′ with a 212-bp PCR product. Primers for mouse gene *Ggta1* were 5′- GTGAACAGCATGAGGGGTTT-3′ and 5′- GTTTTGTTGCCTCTGGGTGT-3′ with a 115-bp PCR product. Primers for mouse gene *Iigp1* were 5′- GGGGCAATAGCTCATTGGTA-3′ and 5′- ACCTCGAAGACATCCCCTTT-3′ with a 104-bp PCR product. Primers for mouse gene *Gbp2* were 5′- GGGGTCACTGTCTGACCACT-3′ and 5′- GGGAAACCTGGGATGAGATT-3′ with a 285- bp PCR product. Primers for mouse gene *Fkbp5* were 5′- TATGCTTATGGCTCGGCTGG-3′ and 5′- CAGCCTTCCAGGTGGACTTT-3′ with a 194-bp PCR product. Primers for mouse gene *Psmb8* were 5′- CAGTCCTGAAGAGGCCTACG-3′ and 5′- CACTTTCACCCAACCGTCTT-3′ with a 121- bp PCR product. Primers for mouse gene *Srgn* were 5′- GCAAGGTTATCCTGCTCGGA-3′ and 5′- TGGGAGGGCCGATGTTATTG-3′ with a 134- bp PCR product. Primers for mouse gene *Amigo2* were 5′- GAGGCGACCATAATGTCGTT-3′ and 5′- GCATCCAACAGTCCGATTCT-3′ with a 263-bp PCR product. Primers for mouse gene *Clcf1* were 5′- CTTCAATCCTCCTCGACTGG-3′ and 5′- TACGTCGGAGTTCAGCTGTG-3′ with a 176-bp PCR product. Primers for mouse gene *Ptx3* were 5′- AACAAGCTCTGTTGCCCATT-3′ and 5′- TCCCAAATGGAACATTGGAT-3′ with a 147-bp PCR product. Primers for mouse gene *S100a10* were 5′- CCTCTGGCTGTGGACAAAAT-3′ and 5′- CTGCTCACAAGAAGCAGTGG-3′ with a 238-bp PCR product. Primers for mouse gene *Sphk1* were 5′- GATGCATGAGGTGGTGAATG-3′ and 5′- TGCTCGTACCCAGCATAGTG-3′ with a 135-bp PCR product. Primers for mouse gene *Cd109* were 5′- CACAGTCGGGAGCCCTAAAG-3′ and 5′- GCAGCGATTTCGATGTCCAC-3′ with a 147- bp PCR product. Primers for mouse gene *Ptgs2* were 5′- GCTGTACAAGCAGTGGCAAA-3′ and 5′- CCCCAAAGATAGCATCTGGA-3′ with a 232-bp PCR product. Primers for mouse gene *Emp1* were 5′- GAGACACTGGCCAGAAAAGC-3′ and 5′- TAAAAGGCAAGGGAATGCAC-3′ with a 183- bp PCR product. Primers for mouse gene *Slc10a6* were 5′- GCTTCGGTGGTATGATGCTT-3′ and 5′- CCACAGGCTTTTCTGGTGAT-3′ with a 217- bp PCR product. Primers for mouse gene *Tm4sf1* were 5′- GCCCAAGCATATTGTGGAGT-3′ and 5′- AGGGTAGGATGTGGCACAAG-3′ with a 258-bp PCR product. Primers for mouse gene *B3gnt5* were 5′- CGTGGGGCAATGAGAACTAT-3′ and 5′- CCCAGCTGAACTGAAGAAGG-3′ with a 207- bp PCR product. Primers for mouse gene *Cd14* were 5′- GGACTGATCTCAGCCCTCTG-3′ and 5′- GCTTCAGCCCAGTGAAAGAC-3′ with a 232- bp PCR product. Primers for mouse *GFAP* variant 1 gene were 5′- GACTATCGCCGCCAACTGCA-3′ and 5′- CTAAGGGAGAGCTGGCAGGG-3′ with a 447-bp PCR product. Primers for mouse *GFAP* variant 2 gene were 5′- GACTATCGCCGCCAACTGCA-3′ and 5′- TCACATCACCACGTCCTTGT-3′ with a 453-bp PCR product.

### Plasmid construction

pFlag-ErbB2, pcDNA3-ErbB3, pFlag-ErbB4 and pcDNA3-EGFR were used in previous reports [77–79]. DnEGFR complementary DNA was amplified from genomic DNA of *TRE*-dnEGFR mice and cloned into pcDNA3.1/myc-His (−)A vector by using cloning sites *Bam*HI and *Xho*I. Constructed plasmid was purified and sequenced. Fused myc-tagged dnEGFR was verified by detecting a band around 105 kDa in lysates of transfected HEK293 cells using anti-myc antibody (9E10) for WB.

### HEK293 cell culture and transfection

HEK293 cells were grown in Dulbecco’s modified Eagle’s medium (DMEM) supplemented with 10% fetal bovine serum (FBS), 50 U/ml penicillin and 50 μg/ml streptomycin, and were transfected using polyethylenimine as previously described [78]. Briefly, cells were cultured in six-well plates to 80% confluence and incubated for 6 h with precipitates formed by 2 μg of plasmid DNA and 2 μl of 0.5% (wt/vol, pH 7.0) polyethylenimine (Sigma-Aldrich, catalog no. 40 872-7). After replacing with fresh medium, cells were cultured in DMEM containing 10% FBS for 24 h before harvesting.

### Primary astrocyte culture and cell growth assay

The cortices of mice at postnatal 2–5 days (P2–P5) were isolated and sheared into pieces, and digested by 0.25% Trypsin in Ca^2+^Mg^2+^-free PBS for 30 min at 37 °C. Trypsinized tissues were triturated and passed through 70 μm mesh. Dissociated cells were collected and washed by DMEM medium once before plating into a poly-l-lysine-coated dish (Φ100 mm) with 10 ml DMEM supplemented with 10% FBS. Culturing media were replaced completely with fresh DMEM plus 10% FBS the next day to remove the dead cells. Remained cells were cultured until reaching 100% confluence with media changed every 2–3 days. Primarily cultured astrocytes could be passaged once at 1 to 3 ratios, and the next generation of astrocytes could be collected when reaching the 100% confluence and frozen into liquid nitrogen. Over 95% of cells in the cultures were positive for GFAP and Acsbg1. When using for measurement of cell growth rate, astrocytes were seeded into a 24-well plate at 5×10^3^ cells per well. Astrocytes in one of the 24 wells were trypsinized and collected every 3 days to count the cell number by hemocytometer. Averaged numbers from three independent experiments were obtained for each time point.

### TUNEL assay

Apoptotic cells were examined with TUNEL assay (Yeasen) according to the manufacturer’s instructions. In brief, brain slices were digested for 10 min by proteinase K (20 μg/ml) at room temperature after IF staining. After washing twice with PBS, brain slices were incubated with equilibration buffer for 30 min at room temperature, and subsequently with Alexa Fluor 488-12-dUTP Labeling Mix for 60 min at 37 °C. After washing with PBS for three times, brain slices were stained with DAPI (2 μg/ml, Roche, Basel, Switzerland) before being mounted under coverslips.

### Statistical analysis

Unless otherwise indicated, data were expressed as mean±s.d. from at least three independent experiments, and analyzed by paired *t*-test by comparing the data between littermates or the same batch of experiments. Statistical significance was considered when *P* is smaller than 0.05.
