# Supplementary material for: A role for ErbB signaling in the induction of reactive astrogliosis
Source: Cell Discov. 2017 Dec 5;3:17044–. doi: 10.1038/celldisc.2017.44 (PMC5717352; doi:10.1038/celldisc.2017.44)
Supplement: Supplementary Figures [file celldisc201744-s1.pdf]

Supplementary Information:

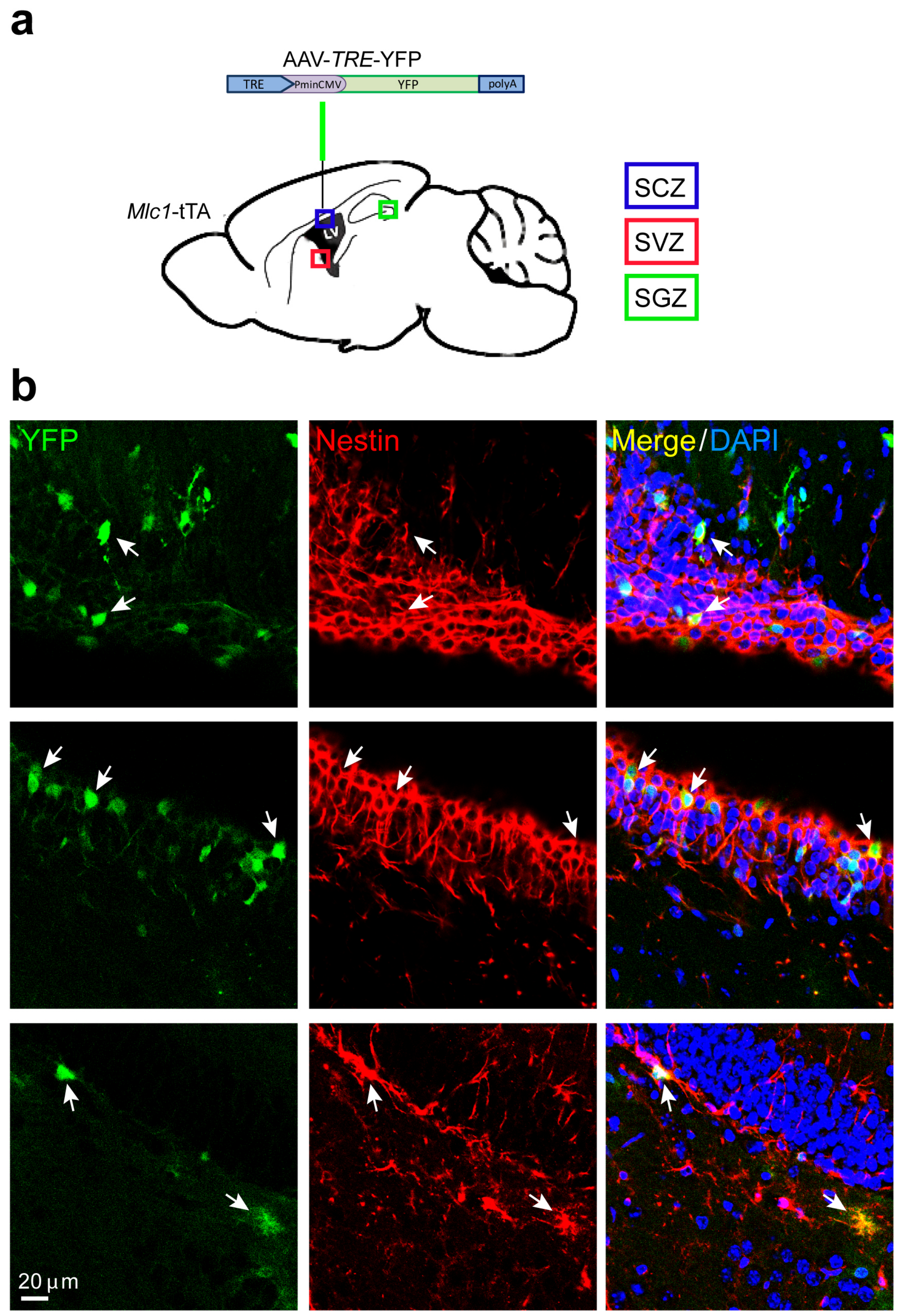

Figure S1 *Mlc1*-tTA targeted neural stem cells strictly in NSC niches. (**a**) Schematic illustration of stereotaxic injection sites for adult neural stem cell (NSC) niches. SCZ, subcorpus callosum zone; SVZ, subventricular zone; SGZ, subgranular zone. (**b**) *Mlc1*-tTA targeted NSCs specifically in NSC niches. Shown are representative images of *TRE*-YFP expression in adult NSCs of *Mlc1*-tTA mice at one month old. AAV-*TRE*-YFP was stereotaxically injected into indicated brain regions. Fixed brains collected one day later were sectioned and immunostained for nestin to label adult NSCs. White arrows, double positive cells.

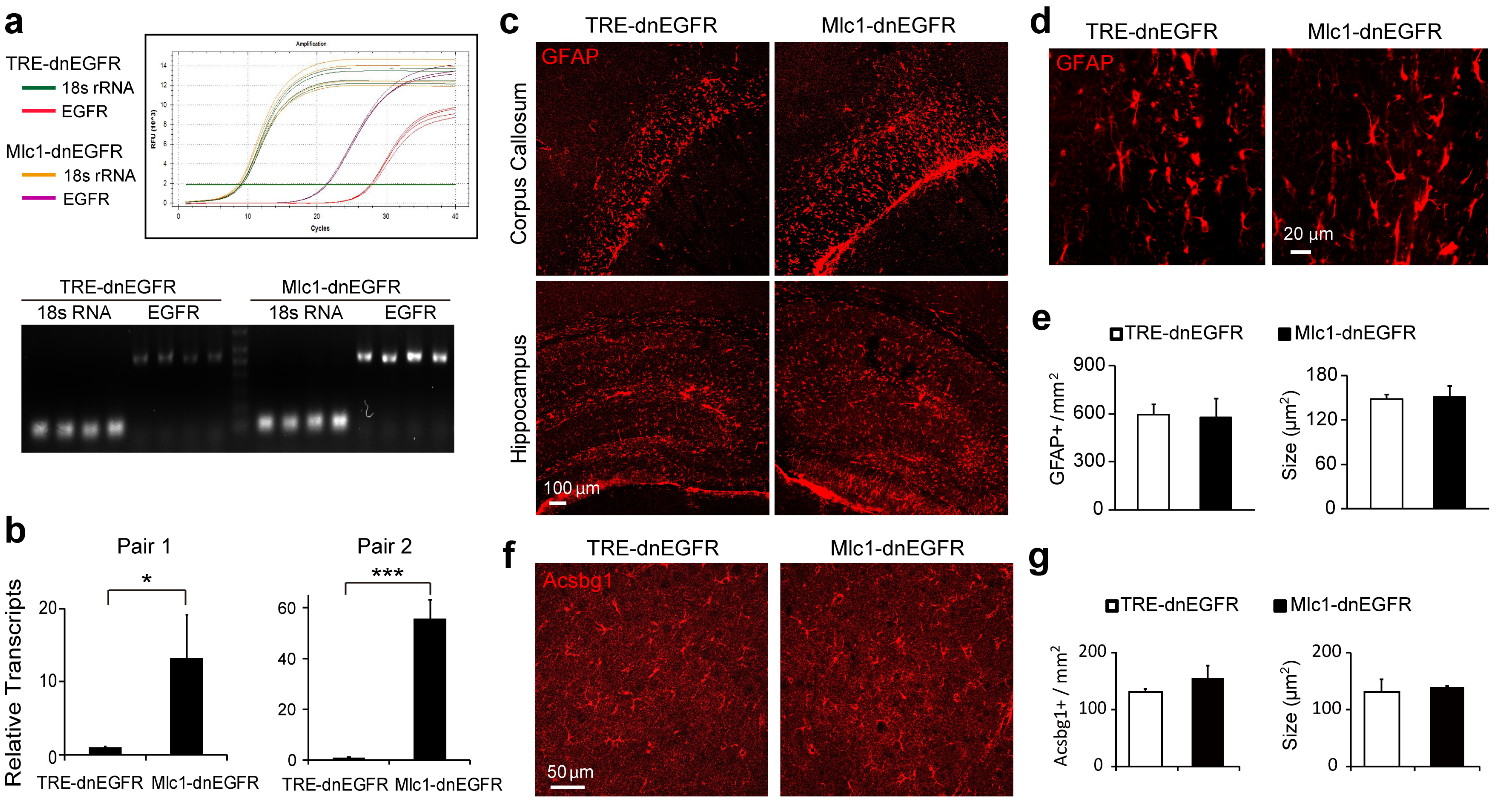

Figure S2 Expression of dnEGFR in astrocytes did not affect astrocyte development. (**a**) Representative real-time RT-PCR results of EGFR/dnEGFR transcripts in cortices from *Mlc1*-dnEGFR and littermate control mice at one month old. Total RNA was extracted from isolated cortices and processed into real-time RT-PCR by using specific primers. Gel image showed the PCR products of four repeats after full run (40 cycles) of real-time RT-PCR. Note the apparent increase of EGFR/dnEGFR PCR products in *Mlc1*-dnEGFR group. (**b**) Quantitative analyses of the transcript increase of dnEGFR in the cortices of *Mlc1*-dnEGFR mice in comparison with that of littermate controls at one month old. Relative transcripts of dnEGFR/EGFR were normalized by that of the internal control 18S rRNA. Shown are results from two separate littermate pairs. *, $P = 0.023$, ***, $P = 0.0008$, $n = 3$ replicates for each pair of mice, unpaired t test. (**c**) Immunostaining of GFAP demonstrated the same astrocyte distribution pattern in the brain of *Mlc1*-dnEGFR and littermate control mice at one month old. (**d**) High power images of GFAP-immunostained corpora callosa of *Mlc1*-dnEGFR and littermate control mice at one month old. (**e**) Quantitative analyses of astrocyte numbers or sizes based on GFAP immunoreactivity in the corpora callosa of *Mlc1*-dnEGFR and littermate mice. $n = 3$ for each group, paired t test. (**f**) Immunostaining of Acsbg1 demonstrated similar astrocyte distributions in the cortices of *Mlc1*-dnEGFR and littermate control mice at one month old. (**g**) Quantitative analyses of astrocyte numbers or sizes based on Acsbg1 immunoreactivity in the cortices of *Mlc1*-dnEGFR and littermate mice. $n = 3$ for each group, paired t test.

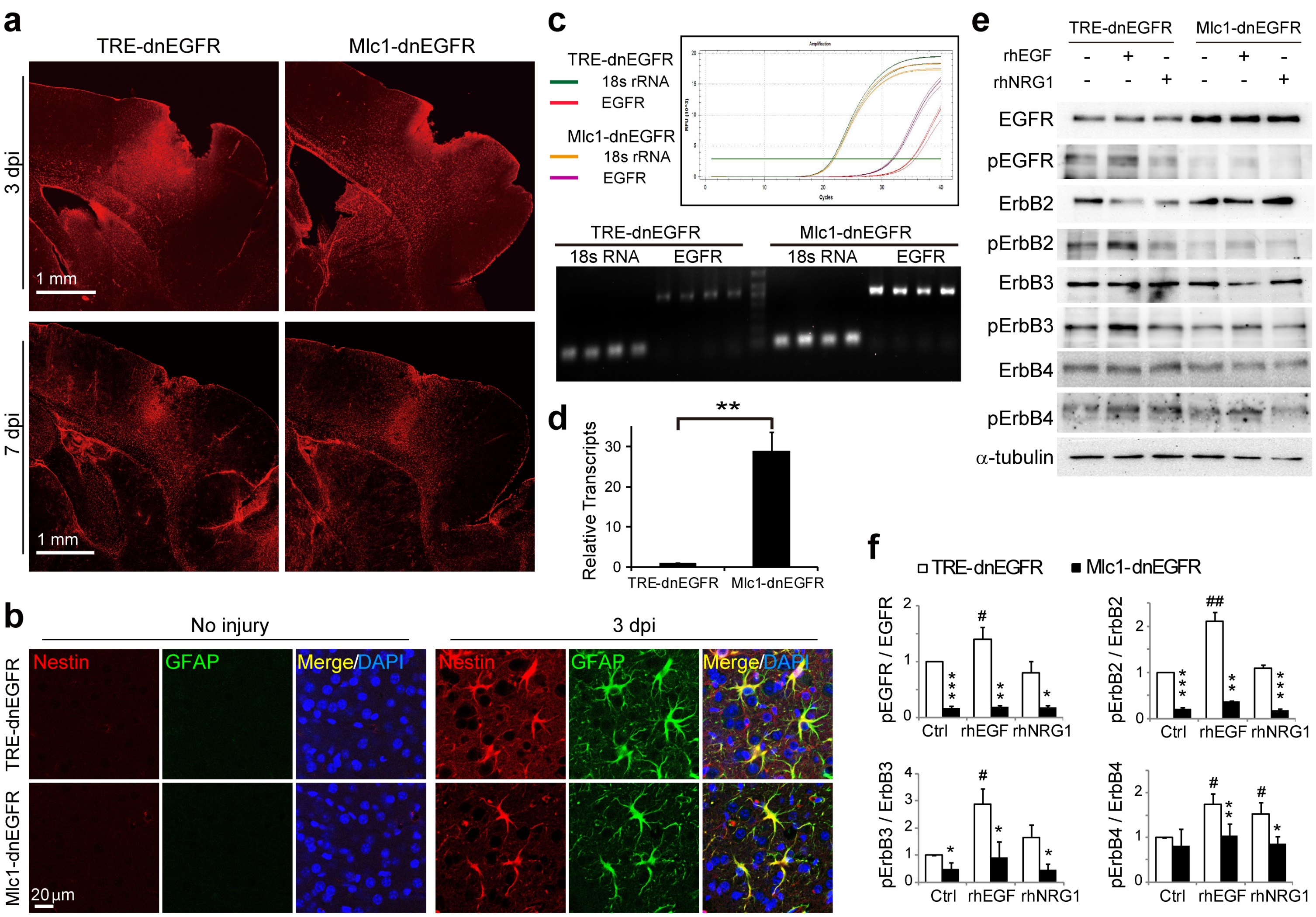

Figure S3 Effects of dnEGFR expression on ErbB receptor activation and molecular hallmarks in reactive astrocytes. (**a**) Astrogliosis was induced by stab wound injury in the cortices of both *Mlc1*-dnEGFR and littermate control mice. Shown are representative GFAP-immunostaining results of injured cortices 3 days or 7 days post injury (dpi). Note the similar increase in GFAP immunoreactivity in the area adjacent to the injury sites in both groups. (**b**) Nestin expressed in reactive cortical astrocyte 3 days post injury. Cortical slices from *Mlc1*-dnEGFR and littermate control mice with or without injury were immunostained for GFAP and nestin. Note there were neither GFAP nor nestin expressing in intact cortices. (**c**) Representative real-time RT-PCR results of EGFR/dnEGFR transcripts in injured cortical tissues of *Mlc1*-dnEGFR and littermate control mice 3 days post injury. Gel image showed the PCR products of four repeats after full run (40 cycles) of real-time RT-PCR. Note the apparent increase of EGFR/dnEGFR PCR products in *Mlc1*-dnEGFR group. (**d**) Quantitative analysis of the transcript increase of dnEGFR in the injured cortical tissues of a *Mlc1*-dnEGFR mouse in comparison with that of littermate control 3 days post injury. **, $P = 0.0012$, $n = 3$ replicates, unpaired *t* test. (**e**) Activities of ErbB receptors in primary astrocytes of *Mlc1*-dnEGFR and control mice induced by ErbB receptor ligands. Primary astrocytes were treated with saline (Ctrl), rhEGF (1 μg/ml), or rhNRG1 (100 ng/ml) for 15 min, respectively, and ErbB receptors and their phosphorylation levels in cell lysates were examined by Western blotting using specific antibodies. (**f**) Quantitative analyses of ErbB phosphorylation levels revealed by Western blotting in astrocytes stimulated by ErbB ligands. Phosphorylation levels of indicated proteins were normalized by its total protein levels. ##, $P < 0.01$; #, $P < 0.05$; as compared with the treatment with saline. ***, $P < 0.001$; **, $P < 0.01$; *, $P < 0.05$; as compared with control astrocytes with the same treatment. $n = 3$ for each protein, paired *t* test.

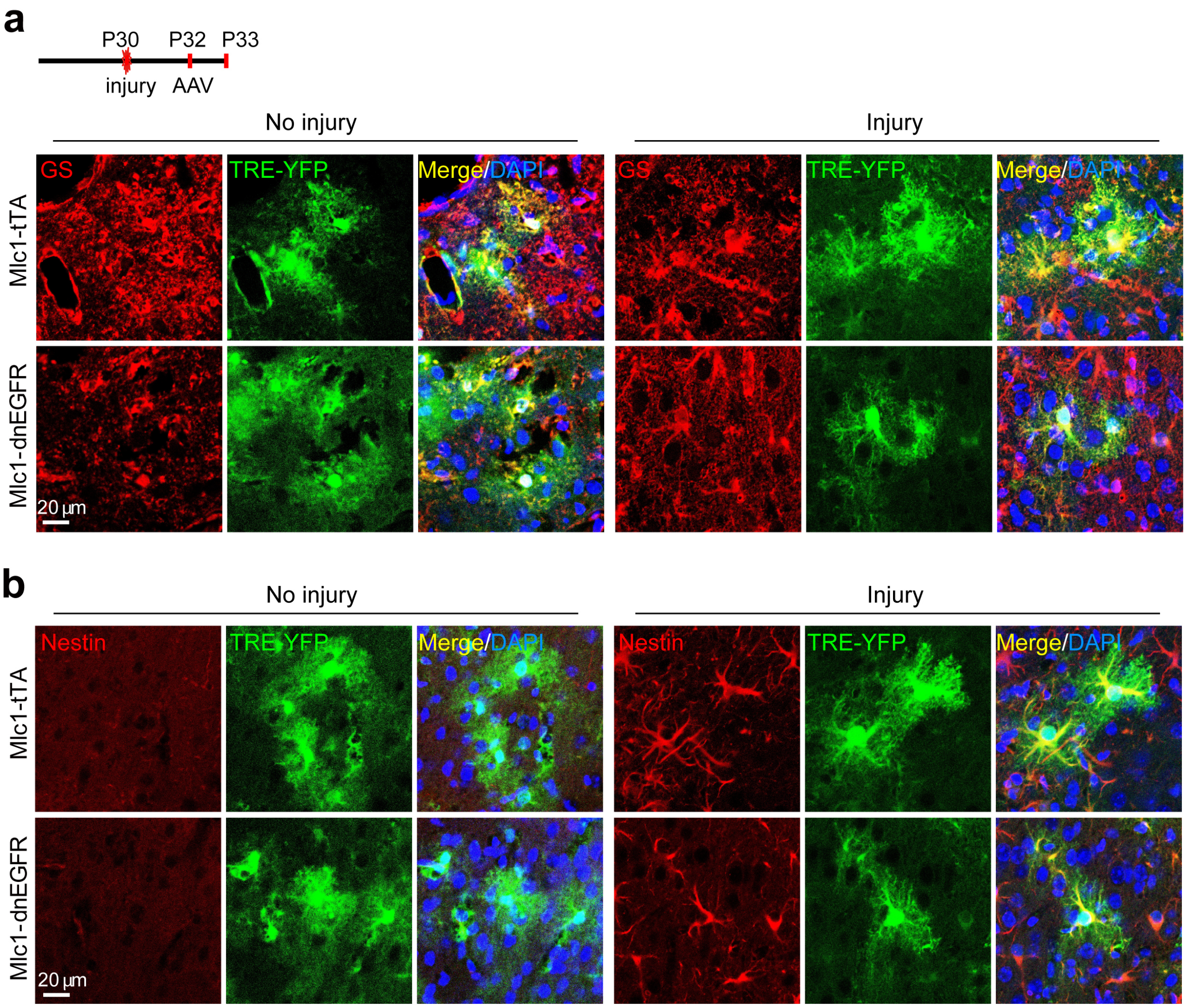

Figure S4 AAV-*TRE*-YFP targeted normal astrocytes in the uninjured cortices and reactive astrocytes in the injured cortices. AAV-*TRE*-YFP labeled cells were immunopositive for astrocyte marker glutamine synthetase (GS) in either the injured or the uninjured cortices (**a**), but only positive for nestin in the injured cortices (**b**), of *Mlc1*-dnEGFR and *Mlc1*-tTA mice. Images were taken under the 40X oil-immersion objective of a Zeiss710 confocal microscope. Note the reactive astrocytes labeled by YFP in the injured cortices of both mice on 3 days post injury (dpi) exhibited bigger sizes than those in the uninjured cortices. Moreover, the reactive astrocytes labeled by YFP in the injured cortices of *Mlc1*-dnEGFR mice were smaller than those in control mice.

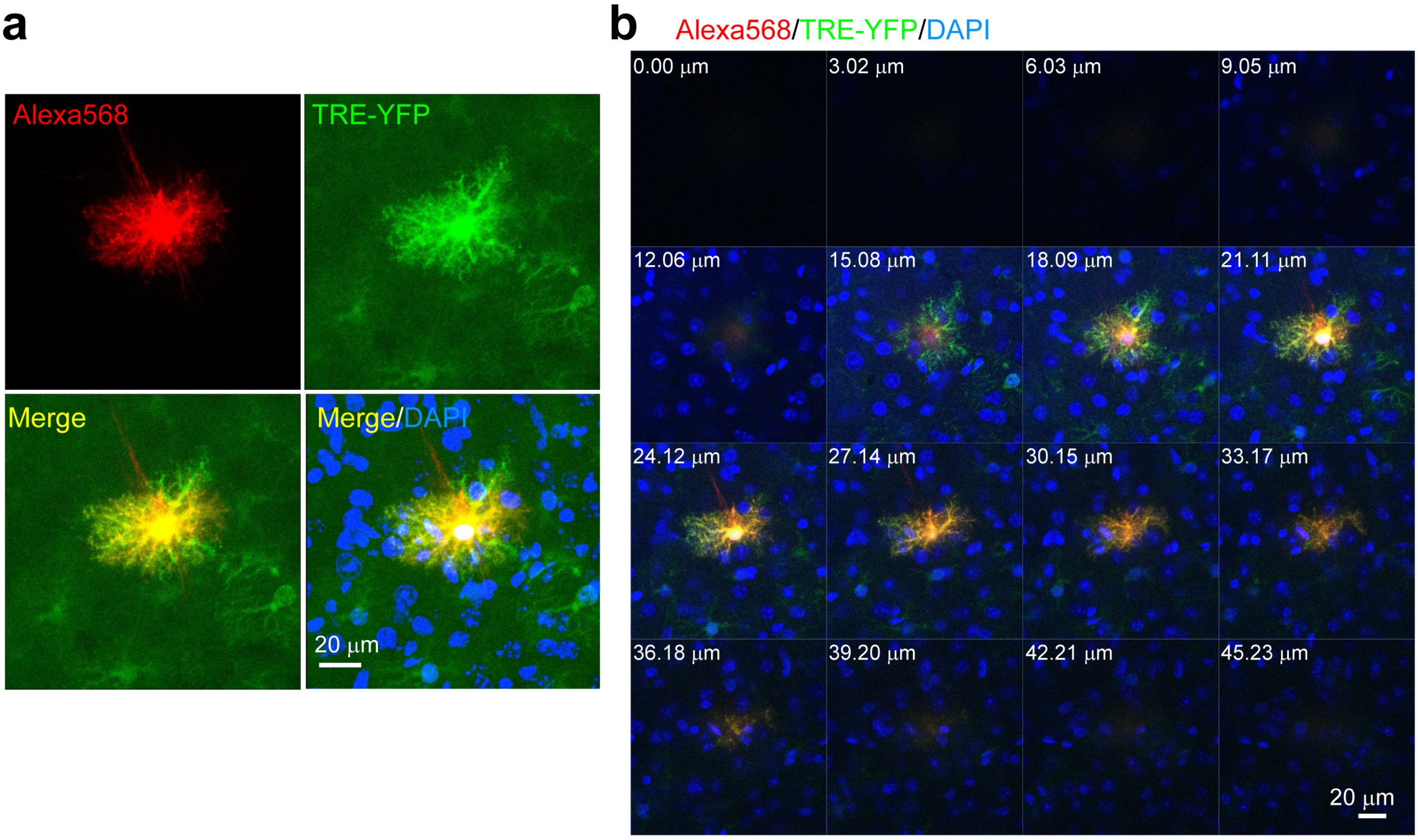

Figure S5 Morphological exhibition of cortical astrocytes by *TRE*-YFP expression. (**a**) Comparison of the labeling of cortical reactive astrocytes by *TRE*-YFP expression and fluorescence dye filling. AAV-*TRE*-YFP labeled astrocytes in injured cortices 3 days post injury were identified under a fluorescence microscope and filled with fluorescence dye Alexa Fluor 568 by glass micropipettes. Shown are projected images of a labeled astrocyte that were captured at Z-stack series by a Zesiss710 confocal microscope with a 40X oil-immersion objective. Note that YFP labeled cell bodies and all fine processes and exhibited a more complete morphology of the cell than the fluorescence dye did. (**b**) Shown is the gallery of Z-stack serial images of the same astrocyte in **a**. Note at every optical slice, YFP exhibited better coverage of the cell than Alexa Fluor 568 did due to labeling of a live cell. Moreover, it was noticeable that the biggest fluorescence area of the astrocyte taken by the confocal microscope was within 6.3-µm Z-axial range through its cell body (from 18.09 µm to 24.12 µm).

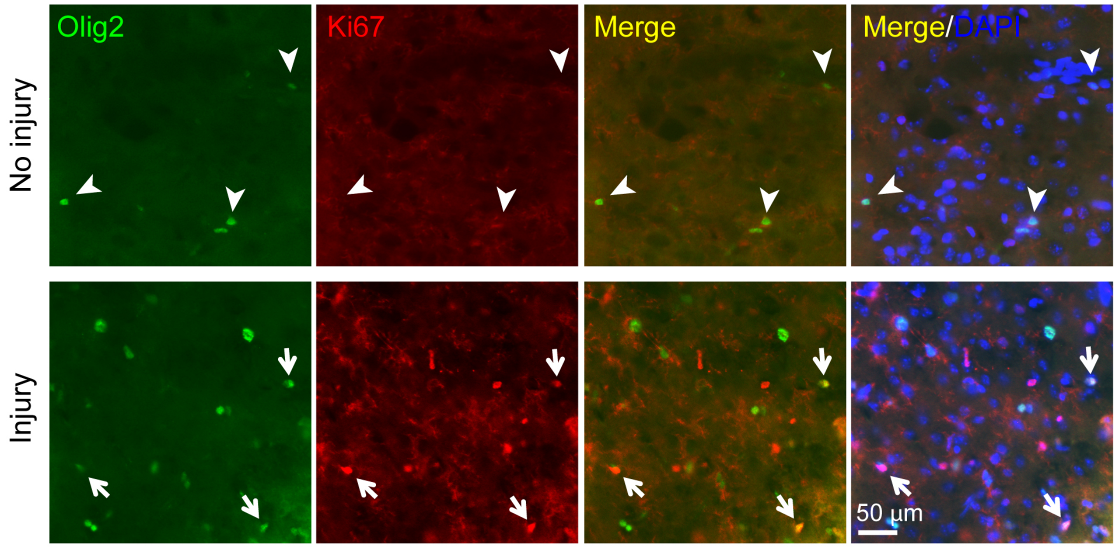

Figure S6 Colocalization of Olig2^{+} and Ki67^{+} nuclei induced by stab wound injury. Uninjured or injured cortices from wild type mice were sectioned and immunostained for Olig2 and Ki67. Note that injury induced the number of Olig2^{+} nuclei as well as Ki67^{+} nuclei increased in cortical regions adjacent to the injury sites. Arrows indicate the colocalization of Olig2 and Ki67 in the same nuclei. Arrowheads indicate the nuclei positive for Olig2 only.

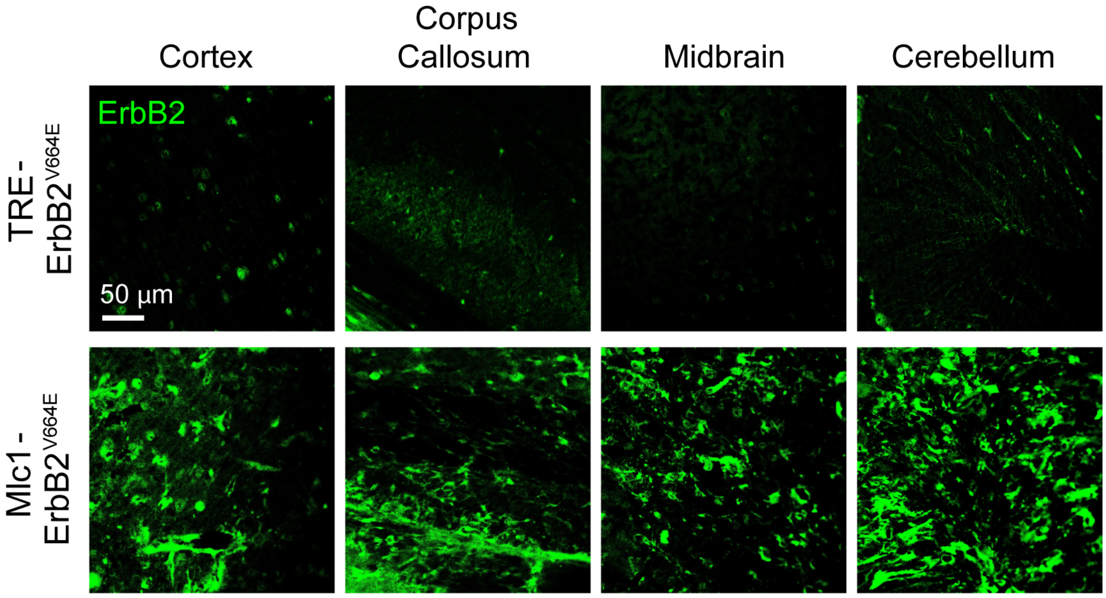

Figure S7 ErbB2^{V664E} was expressed in various brain regions in *Mlc1*-ErbB2^{V664E} mice. Brain slices from *Mlc1*-ErbB2^{V664E} and littermate control mice 20 days after Dox withdrawal were immunostained for ErbB2. Note ErbB2^{+} cells increased dramatically in different brain regions of *Mlc1*-ErbB2^{V664E} mice as compared with that of littermate controls.

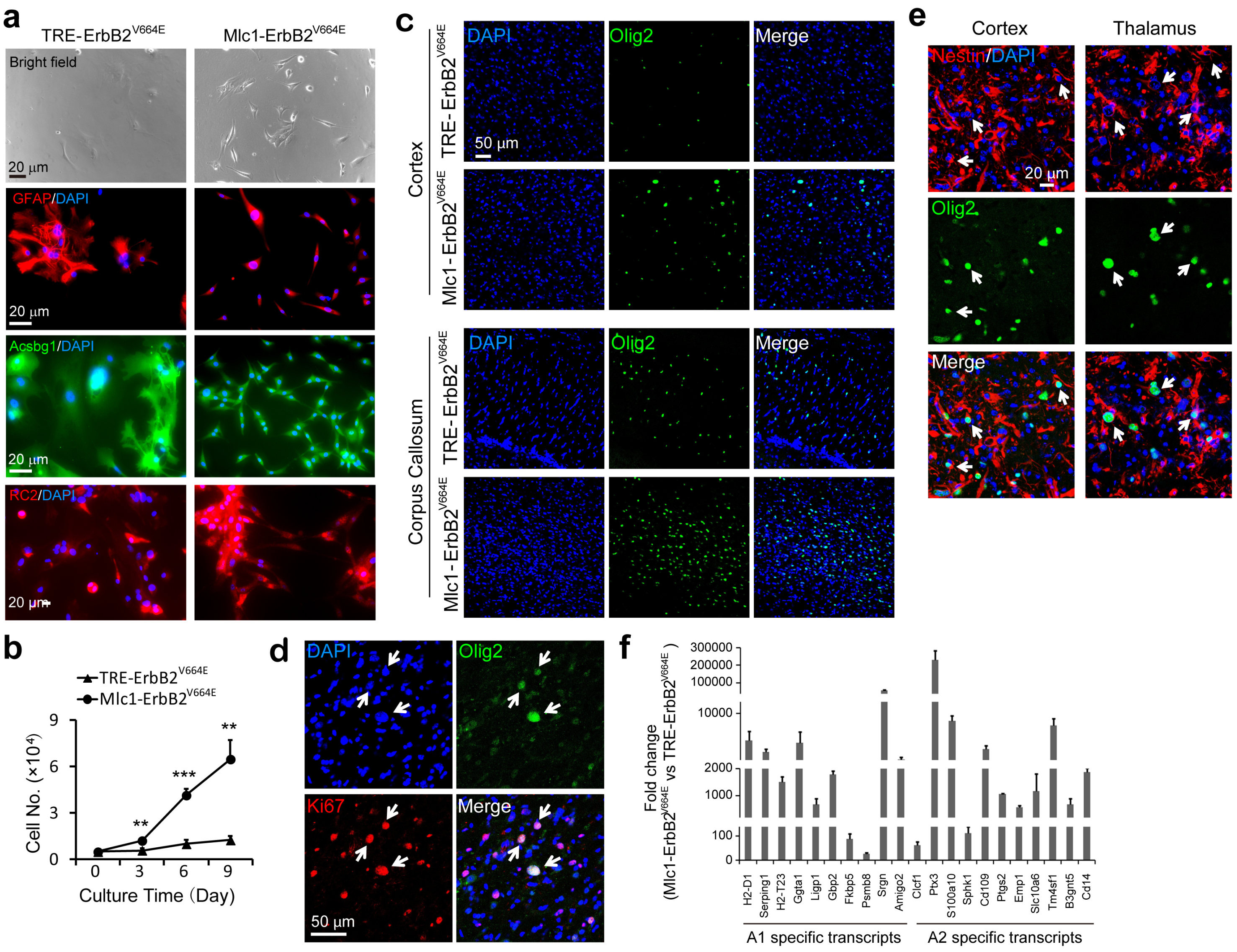

Figure S8 Cell-autonomous activation of ErbB signaling in astrocytes promoted cell proliferation. (**a**) Primarily cultured astrocytes from *Mlc1*-ErbB2^{V664E} brain exhibited different morphology from that of control mice. Shown are representative images of cultured astrocytes in bright field, or immunostained for GFAP, Acsbg1, or RC2. Note normal astrocytes cultured *in vitro* were flat and adhered well to the dish bottom, while astrocytes isolated from *Mlc1*-ErbB2^{V664E} brain were plump and small. (**b**) Cell growth rate of cultured astrocytes from *Mlc1*-ErbB2^{V664E} mice was much faster than that from control mice. Primary cultured astrocytes were plated into 24-well plate at 5×10^3 cells per well, and cells in each well were trypsinized and counted at different time points. **, $P < 0.01$; ***, $P < 0.001$; $n = 3$ replicates for each time point, one-way ANOVA. (**c**) Increased Olig2$^+$ nuclei in the brain of *Mlc1*-ErbB2^{V664E} mice. Brain slices from *Mlc1*-ErbB2^{V664E} and littermate control mice 20 days after Dox withdrawal were immunostained for Olig2. (**d**) Colocalization of Olig2 with Ki67 in some nuclei in the cortices of *Mlc1*-ErbB2^{V664E} mice. White arrows, Ki67$^+$Olig2$^+$ nuclei. (**e**) Localization of Olig2$^+$ nuclei in nestin$^+$ cells in the brain of *Mlc1*-ErbB2^{V664E} mice. White arrows, nestin$^+$Olig2$^+$ cells. A nucleus (DAPI$^+$) for a nestin$^+$ cell was identified by the association with its main cell body. (**f**) Reactive astrocytes in *Mlc1*-ErbB2^{V664E} mice exhibited gene expression characteristics of both A1 and A2 subtypes. Total RNA extracted from the cortices of *Mlc1*-ErbB2^{V664E} and control mice were subjected to real-time RT-PCR with specific primers to examine the characteristic genes of A1 or A2 subtypes. Results shown were normalized by the mRNA levels of internal control *gapdh* and the same genes in control mice. $n = 3$ replicates for each gene.

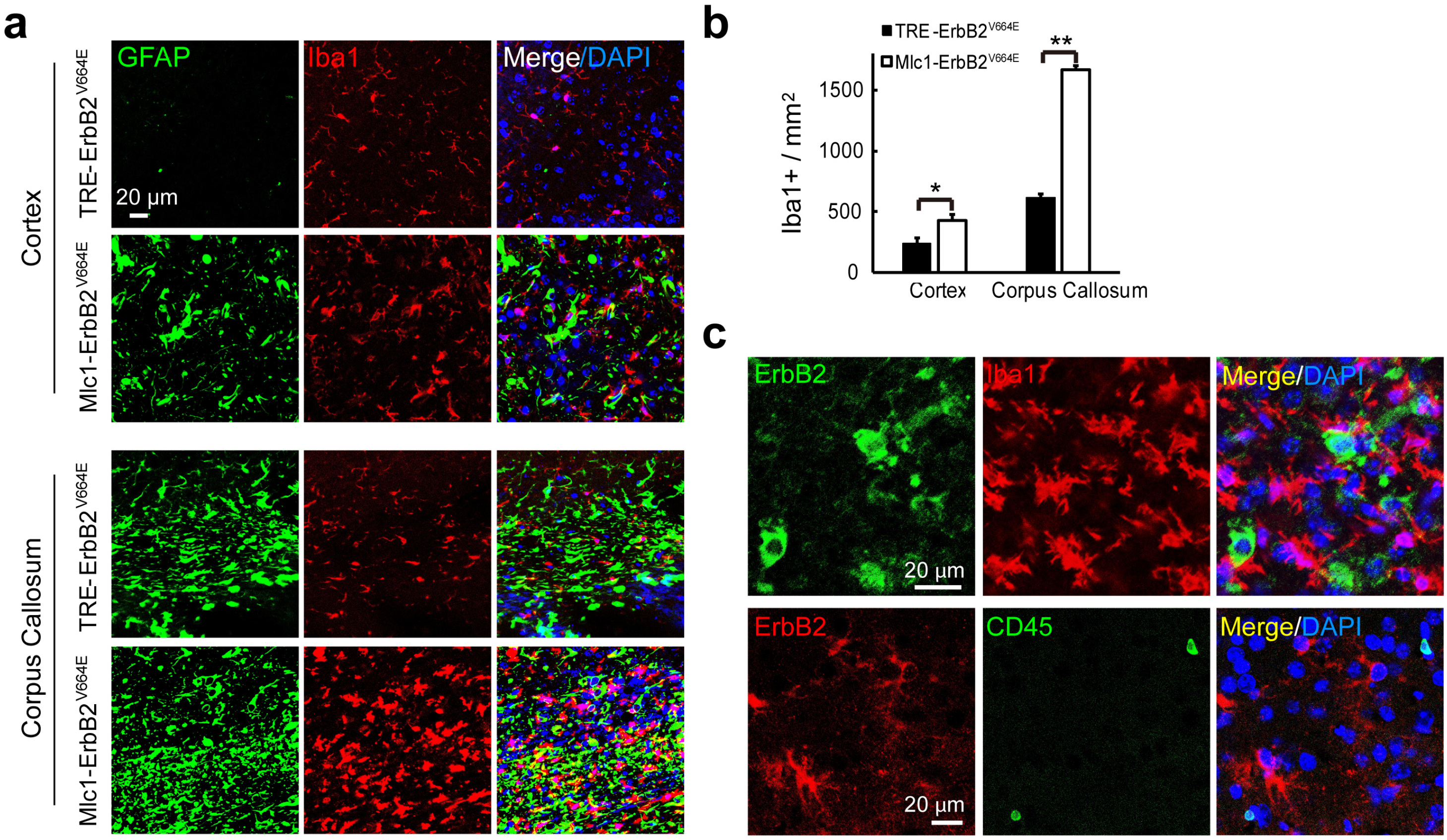

Figure S9 Spontaneous astrogliosis induced by ErbB activation in astrocytes was accompanied by inflammation throughout the brain. (**a**) Reactive microglia was induced in the brain of *Mlc1*-ErbB2^{V664E} mice. Shown are representative immunostaining results of GFAP and microglia marker Iba1 in brain slices of *Mlc1*-ErbB2^{V664E} and littermate control mice 20 days after Dox withdrawal. (**b**) Quantitative analyses of Iba1^{+} cell densities in the cortices and corpora callosa in *Mlc1*-ErbB2^{V664E} mice and littermate controls. *, $P = 0.0287$; **, $P = 0.0007$; $n = 3$ for each group, paired t test. (**c**) No expression of ErbB2^{V664E} in reactive microglia or leukocytes of *Mlc1*-ErbB2^{V664E} mice. Shown are representative immunostaining results of ErbB2 and Iba1, or ErbB2 and CD45, in the cortices of *Mlc1*-ErbB2^{V664E} mice after Dox withdrawal.

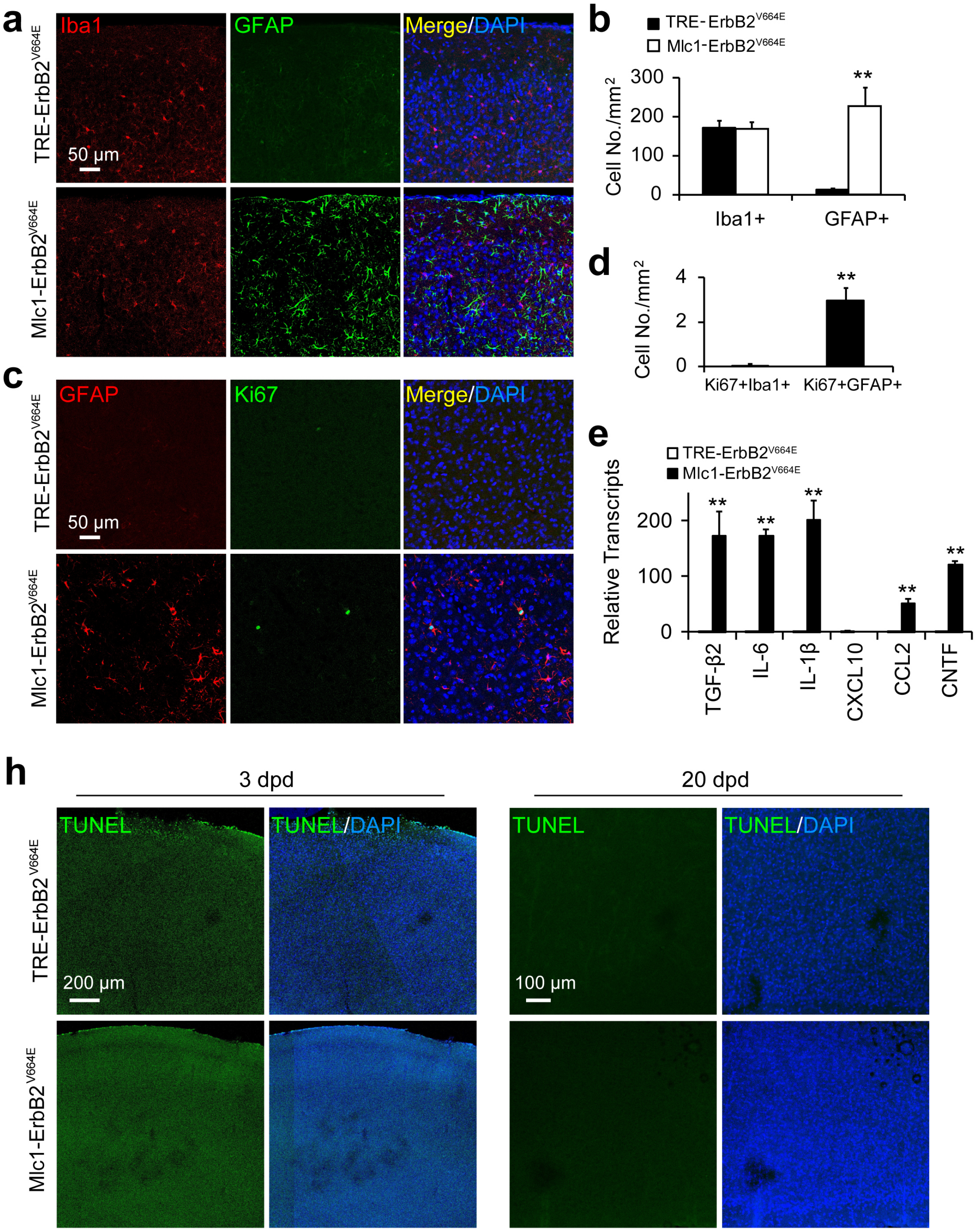

Figure S10 Inflammation in the brain of *Mlc1*-ErbB2^{V664E} mice was induced by reactive astrocytes. (**a**) Iba1^{+} cells were not reactive in the brain of *Mlc1*-ErbB2^{V664E} mice with early astrogliosis. Shown are representative immunostaining results of GFAP and Iba1 in cortical slices of *Mlc1*-ErbB2^{V664E} and littermate control mice 3 days after Dox withdrawal. Note that cortical astrocytes already started to express GFAP as this stage. (**b**) Quantitative analyses of Iba1^{+} and GFAP^{+} cell densities in the cortices of *Mlc1*-ErbB2^{V664E} mice and littermate controls 3 days after Dox withdrawal. **, $P = 0.001$, $n = 3$ for each group, paired t test. (**c**) Only GFAP^{+} cells in the brain of *Mlc1*-ErbB2^{V664E} mice with early astrogliosis had proliferation marker Ki67. Shown are representative immunostaining results of GFAP and Ki67 in cortical slices of

Mlc1-ErbB2^{V664E} and littermate control mice 3 days after Dox withdrawal. Note the few Ki67^{+} nuclei identified in *Mlc1*-ErbB2^{V664E} cortex localized well in GFAP^{+} cells. (**d**) Quantitative analyses of Ki67^{+}Iba1^{+} and Ki67^{+}GFAP^{+} cell densities in the cortices of *Mlc1*-ErbB2^{V664E} mice 3 days after Dox withdrawal. $n = 3$ for each group; **, $P = 0.006$, as compared with the numbers of Ki67^{+}Iba1^{+} cells. (**e**) Cytokine production increased in astrocytes from *Mlc1*-ErbB2^{V664E} mice. Total RNA extracted from primary astrocytes from control and *Mlc1*-ErbB2^{V664E} mice were subjected to real-time RT-PCR with specific primers to CNTF, CCL2, CXCL10, IL-1β, IL-6, and TGF-β2. (**f**) No cell apoptosis was induced in the brain of *Mlc1*-ErbB2^{V664E} mice by Dox withdrawal. Shown are TUNEL assay results of the cortical slices from *Mlc1*-ErbB2^{V664E} and littermate control mice 3 or 20 days post Dox withdrawal (dpd).

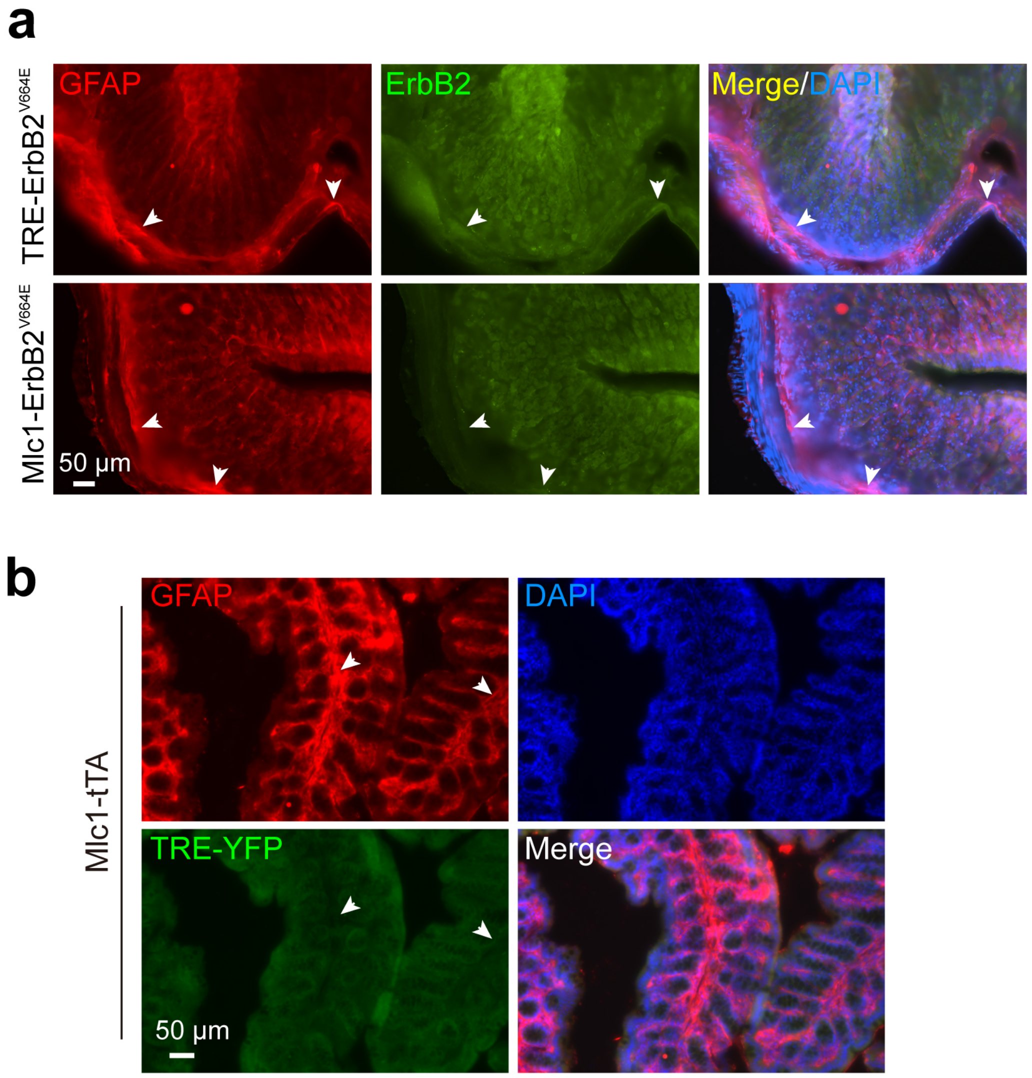

Figure S11 *Mlc1*-tTA did not target enteric glia in the gastrointestinal tract. (**a**) ErbB2 was not overexpressed in gastrointestinal tract of *Mlc1*-ErbB2^{V664E} mice. Stomach sections from *Mlc1*-ErbB2^{V664E} or control mice 20 days after Dox withdrawal were immunostained for GFAP and ErbB2. Enteric glia were labeled by GFAP immunostaining. Arrowheads indicate the cells positive for GFAP but not ErbB2. (**b**) AAV-*TRE*-YFP did not label cells in gastrointestinal tract of *Mlc1*-tTA mice. Small intestine of *Mlc1*-tTA mice were exposed and 2 µl AAV-*TRE*-YFP was injected into the intestine wall. One day later the injected part was isolated and sectioned for immunostaining of GFAP. Enteric glia in the submucosa of plicae circulares were positive for GFAP (arrowheads). No YFP signal was detected in the virus-injected intestine sections. There were some spots in the sections exhibited higher green autofluorescence due to tissue folding or overlapping at the edge.

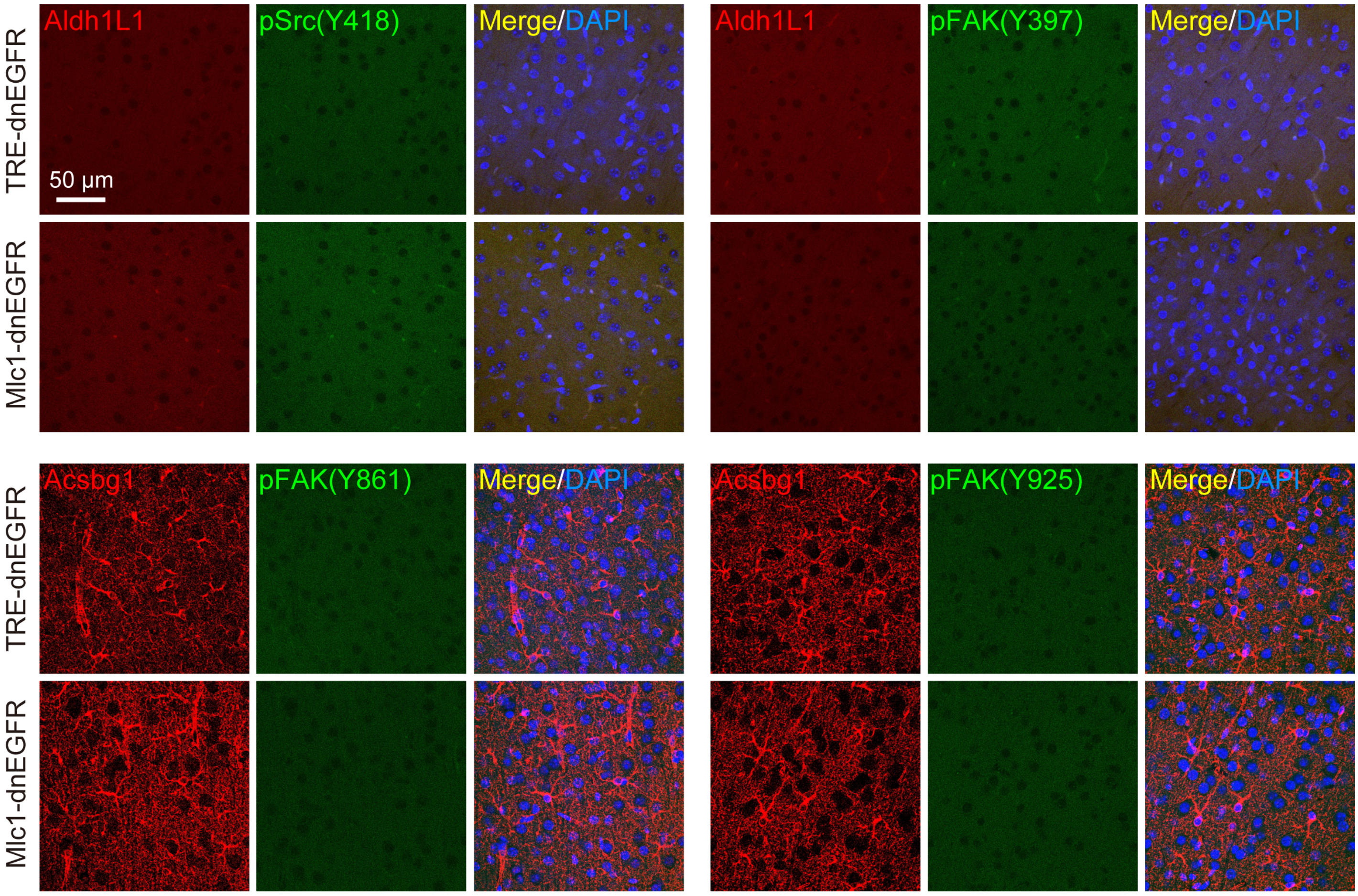

Figure S12 No detectable Src and FAK activities in the uninjured cortices by immunostaining. Cortical slices from uninjured brains of *Mlc1*-dnEGFR and littermate control mice were co-immunostained by mouse antibodies against Aldh1L1 or Acsbg1, both are previously reported markers for cortical astrocytes, and rabbit/goat antibodies against the active forms of FAK or Src, respectively. Although the antibody against Aldh1L1 failed to label cortical astrocytes, the immunostaining results showed there were neither FAK nor Src activities detected in normal astrocytes of either *Mlc1*-dnEGFR or control mice.
